# Inflammatory biotype of ADHD is linked to chronic stress: a data-driven analysis of the inflammatory proteome

**DOI:** 10.1038/s41398-023-02729-3

**Published:** 2024-01-18

**Authors:** Isabel Schnorr, Anne Siegl, Sonja Luckhardt, Söri Wenz, Hendrik Friedrichsen, Hiba El Jomaa, Annebirth Steinmann, Tünde Kilencz, Gara Arteaga-Henríquez, Carolina Ramos-Sayalero, Pol Ibanez-Jimenez, Silvia Karina Rosales-Ortiz, István Bitter, Christian Fadeuilhe, Marc Ferrer, Catharina Lavebratt, János M. Réthelyi, Vanesa Richarte, Nanda Rommelse, Josep Antoni Ramos-Quiroga, Alejandro Arias-Vasquez, Eduard Resch, Andreas Reif, Silke Matura, Carmen Schiweck

**Affiliations:** 1 Goethe University Frankfurt, University Hospital, Department of Psychiatry, Psychosomatic Medicine and Psychotherapy, Frankfurt, Germany; 2 Fraunhofer Institute for Translational Medicine and Pharmacology ITMP, Frankfurt, Germany; 3 Semmelweis University, Department of Psychiatry and Psychotherapy, Budapest, Hungary; 4 Department of Mental Health, Hospital Universitari Vall d´Hebron, Barcelona, Catalonia Spain; 5 Biomedical Network Research Center on Mental Health (CIBERSAM), Barcelona, Catalonia Spain; 6 NCRR-The National Center for Register-Based Research, Aarhus University, Aarhus, Denmark; 7 Department of Psychiatry and Forensic Medicine, Universitat Autónoma de Barcelona, Barcelona, Catalonia Spain; 8 Department of Molecular Medicine and Surgery, Karolinska Institutet, and Center for Molecular Medicine, Karolinska University Hospital Solna, Stockholm, Sweden; 9 Department of Human Genetics, Radboud University Medical Center, Donders Institute for Brain, Cognition and Behavior, Nijmegen, The Netherlands; 10 Department of Psychiatry, Radboud University Medical Center, Donders Institute for Brain, Cognition and Behavior, Nijmegen, The Netherlands

**Keywords:** ADHD, Diagnostic markers, Human behaviour

## Abstract

The association between Attention Deficit Hyperactivity Disorder (ADHD) and low-grade inflammation has been explored in children but rarely in adults. Inflammation is characteristic of some, but not all, patients with ADHD and might be influenced by ADHD medication but also lifestyle factors including nutrition, smoking, and stress. It is also still unclear if any specific symptoms are related to inflammation. Therefore, we assessed 96 inflammatory proteins in a deeply phenotyped cohort of 126 adult ADHD participants with a stable medication status using OLINK technology. A data-based, unsupervised hierarchical clustering method could identify two distinct biotypes within the 126 ADHD participants based on their inflammatory profile: a higher inflammatory potential (HIP) and a lower inflammatory protein potential (LIP) group. Biological processes that differed strongest between groups were related to the NF-κB pathway, chemokine signaling, IL-17 signaling, metabolic alterations, and chemokine attraction. A comparison of sample characteristics revealed that the HIP group was more likely to have higher levels of chronic stress (*p* < 0.001), a higher clinical global impression scale score (*p* = 0.030), and a higher risk for suicide (*p* = 0.032). Medication status did not influence protein levels significantly (*p* ≥ 0.074), but psychotropic co-medication (*p* ≤ 0.009) did. In conclusion, our data suggest the presence of two distinct biotypes in adults with ADHD. Higher levels of inflammatory proteins in ADHD are linked to higher levels of chronic perceived stress in a linear fashion. Further research on inflammation in adults with ADHD should take stress levels into account.

## Introduction

Attention deficit hyperactivity disorder (ADHD) is a heterogeneous neurodevelopmental syndrome characterized by behavioral manifestations such as impulsivity, inattention, and hyperactivity [1]. Prevalence of ADHD is around 6% during childhood and 4% during adulthood [2, 3]. While ADHD, per definition, starts before the 12th birthday, it persists into adulthood in up to 60% of patients, although a shift in symptoms can be observed [4]. Externalizing symptoms such as fidgeting and talking excessively attenuate, while internalizing symptoms (e.g., particularly restlessness, impulsivity, and inattention) prevail in adults with ADHD (aADHD) [4]. Pharmacological treatment of ADHD is effective in ~70–80% [5], but the pathophysiological mechanisms leading to ADHD are still incompletely understood. Next to an imbalance of the neurotransmitters dopamine (DA) and noradrenaline (NA) [6, 7], genetic [8], environmental and etiopathological factors [9], recent literature suggests (neuro-)inflammation and defective immune regulation as potential contributing factors to ADHD [10, 11].

The current evidence for (neuro)inflammatory processes in ADHD compared to other neuropsychiatric disorders is sparse but nevertheless intriguing. As such, it is well established that exposure to maternal immune activation in utero is associated with neurodevelopmental disorders mediated by inflammatory cell signaling pathways and epigenetic mechanisms [12]. Large cohort studies showed that children of mothers with an autoimmune disorder were more likely to be diagnosed with ADHD [13]. It stands to reason that inflammation in adulthood may be irrelevant if in utero exposure is sufficient for disease manifestation and can no longer be prevented. However, if altered immune signaling contributes to or maintains ADHD pathology, it may be a valuable target for add-on treatment strategies. It should also be noted that peripheral inflammation is not (necessarily) indicating neuroinflammation. Peripheral inflammation can, however, affect the central nervous system. For example, some cytokines can cross the blood-brain barrier, induce the blood-brain barrier’s endothelial cells to secrete cytokines into the CNS [14], and/or contribute to a more permeable blood-brain barrier [15]. Externally induced inflammation (e.g., during immunotherapy) can alter DA availability [16], influence neural circuits and affect cognition and behavior [17, 18]. A small but increasing body of evidence seems to suggest that peripheral inflammatory proteins are altered in children with ADHD compared to healthy controls. The most recent review, including ten studies with 1046 patients and 3333 controls, suggested a modest increase of Interleukin (IL)-6 in patients with ADHD [19], but reported no change for the majority of proteins (i.e., C-reactive Protein (CRP), IL1-β, IL-10, INF-γ), and even found lower levels for Tumor necrosis factor (TNF)-α. Interestingly, the increase of IL-6 was more prominent in young participants and those who were not medicated. However, sensitivity analyses showed that results hovered at the brink of significance when excluding single studies, warranting caution in generalizing these findings to all patient groups.

Only a few studies have investigated the association of cytokines at the behavior or symptom/subtype level, with mixed results: various cytokines were associated with either behavioral subtype. To illustrate, Oades and colleagues [20] found that IL-13 and IL-16 levels correlated positively with inattention and hyperactivity, respectively. In contrast, Cortese et al. [21] found a significant positive correlation between IL-6 and TNF-α with hyperactive-impulsive ADHD in children and adolescents with ADHD and obesity. Others found that TNF-α levels correlated positively with inattention symptoms [22]. Together, these results have so far been unable to establish a clear association between ADHD-specific behavior and inflammatory proteins.

Several study design issues likely contribute to the above studies’ large heterogeneity and mixed results. First, overall study samples are mostly small, ranging from 40 to 120 participants, and have heterogeneous characteristics, including children and adults with varying levels of impairment and diagnostic procedures, as well as medication status. Second, most studies have been conducted in children, and some pool results of children and adults. During childhood and puberty, the immune system is in a state of transition, amongst others, due to changing levels of steroids and sex hormones throughout adolescence [23, 24]. This makes immune signatures in childhood/puberty and adulthood difficult to compare with adult patients. To our knowledge, up to date, only limited studies in adults exist regarding inflammatory proteins [25–28]. A recent positron emission tomography study [29] in 24 psychotropic-naïve adult patients and controls also pointed out that neuro-inflammatory processes may still be relevant in adulthood: In their study, the authors demonstrated higher microglial activation in the dorsolateral prefrontal cortex and orbitofrontal cortex in participants with ADHD compared to healthy controls, potentially indicating neuro-inflammatory activity. Assessing peripheral cytokines in an adult population with ADHD is also very important for treating comorbid mental disorders such as depression and bipolar disorder since both are highly prevalent in ADHD [30, 31] and have been independently associated with inflammation [32, 33]. Furthermore, it is conceivable that lifestyle factors such as unhealthy diet [34], smoking status [35], and increased stress levels [36] can lead to inflammation and should be taken into account.

Lastly, and importantly, the effect of medication has rarely been explored systematically. The established treatment of choice for ADHD is central nervous system stimulants, primarily methylphenidate (MPH) and amphetamines [e.g., lisdexamfetamine (LDX)] [37]. Both are thought to increase synaptic DA and NA levels [38]. However, next to their primary effect on DA and NA, amphetamines/LDX and MPH can also influence inflammatory proteins by their action on DA or NE receptors and DA-related proteins, which are expressed on many immune cells. In animal models, it was shown that especially relatively higher MPH doses (5 mg/kg/day) could negatively affect brain tissue, leading to microglial activation, neuroinflammation, and neurodegeneration [39–41]. At the same time, low doses (1.5 mg/kg/day) in rodent models of ADHD led to a more balanced immune system and behavior [41]. Findings of rodent and human studies are, of course, not directly comparable, but reports in humans investigating the effect of medication are sparse. Oades et al. [20] found children with ADHD who were medicated to have lower levels of IFN-γ and IL-13, while Misiak and colleagues found IL-6 to be higher in unmedicated children [19]. However, since these cytokines have not been consistently tested in all studies, it is essential to study the effect of medication on cytokine levels.

To address the above issues, we here investigate meticulously sampled, well-characterized participants with aADHD who were either unmedicated or medicated with a stable dose of ADHD medication for at least four weeks. The aim of this study is two-fold: firstly, we conducted an exploratory, data-driven cluster analysis on a wide range of inflammatory proteins to assess which sample characteristics (if any) are associated with heightened inflammatory proteins in ADHD. For this part, we explored demographic characteristics, ADHD subtypes, chronic stress, and additional psychiatric characteristics such as current Major Depressive Episode (MDE), suicidality risk, comorbid Borderline Personality Disorder (BPD), and the Clinical Global Impression Severity scale. Secondly, given the potentially important effect of medication on ADHD, we conducted specific analyses between medicated and unmedicated groups. Here, we hypothesized lower levels of inflammatory cytokines in medicated people with ADHD.

## Methods

### Participants and procedure

Data for this paper was collected between 2019 and 2021 and derived from an extensively characterized cohort of adult patients with ADHD from the multi-center PROBIA study [42].

### Participants

Analyses on cytokine levels and the effect of medication on them in ADHD were conducted on data and material collected during the “PROBIA” study (Clinical Trial Registration: https://classic.clinicaltrials.gov/ct2/show/NCT03495375). Ethical approval was obtained from each study center (Ethical Approval Frankfurt: 269/18; Budapest: 44101-1/2018/EKU; Barcelona: 311/2018). The PROBIA study aimed to address the effect of a synbiotic intervention on irritability (for details, see NCT03495375). For the purpose of this study, only baseline data of participants with ADHD was used. Prospective participants were recruited via advertising and hospital outpatient clinics. Participants had to be between 18 and 65 years of age, with no major neurological, cardiovascular, endocrine, pulmonary, or gastrointestinal illness nor any major psychiatric disorders with psychotic symptoms present in history or at screening. Participants with conditions related to inflammation and/or anti-inflammatory medication were permitted in the study as long as they were stable. Medication was recorded, and a supplemental analysis was performed, excluding those with medication for inflammatory disorders. Importantly, successful inclusion required stable medication for ADHD (e.g., type and stable dosage for at least 30 days, or alternatively, no medication for at least 30 days). Subjects were excluded if they were undergoing immunosuppression or if antibiotics, or probiotics were currently prescribed or taken within the last 30 days. All participants in the present study had to meet DSM-5 criteria for ADHD confirmed by a structured diagnostic interview [ADHD: Diagnostic Interview for Adult ADHD (DIVA 2-0) [43], see below; Additionally, any BPD was assessed with the Structured Clinical Interview for DSM-IV (SCID-II) [44]]. All participants in the study had to have at least moderate illness severity as judged by the Clinical Global Impression Scale (CGI-S) ≥ 4 for study inclusion [45]. Irritability was measured by the self-reported Affective Reactivity Index scale (ARI-S) [46]. Participants were excluded if ADHD diagnosis was not confirmed, or the participant did not reach a score of ≥ 4 in CGI-S or ≥ 5 in ARI-S. At screening, informed consent was obtained prior to any study-related activity. Participants’ characteristics and demographics, such as sex, age, ethnicity, highest education level, tobacco use, salary per month, and current medication status, were documented. Additionally, nutrition intake was collected at each site, but with different methods: in Frankfurt a 3-day online dietary protocol (*myfood24*) was collected at baseline, whereas three 24 hour recall food frequency questionnaires were used in Barcelona and Budapest. From these protocols and questionnaires, we derived details on macro and micronutrients such as total energy consumption, protein, fat, fiber, omega 3/omega 6 ratio, saturated fatty acids, alcohol, and different vitamins. However, since the methods used were strikingly different, they were not directly comparable between sites and thus only served descriptive purposes. The structured DIVA-2.0 [43] interview was performed by trained staff to assess ADHD symptoms during childhood (between the ages of 5–12) and adulthood. At least 6 of 9 attention deficit criteria, as well as 6 of 9 hyperactivity/impulsivity during childhood and adulthood, had to be reached to be enrolled as a participant with confirmed ADHD. A lifelong impairment caused by their symptoms in at least two life situations had to be reported as part of the structured interview. Partners or family members were not interviewed in the scope of the study. Current ADHD symptoms severity was assessed via the self-reported ADHD Rating Scale (ADHD-RS) [47]. The 18 items with a score ranging from 0–54 assess 9 in inattention, 5 hyperactivity, and 4 impulsivity symptoms in the last six months. Psychiatric comorbidities such as current MDE and Suicidality risk were assessed with the Mini International Neuropsychiatric Interview, DSM-IV (M.I.N.I.) [48]. IQ was assessed with the WAIS-III or WAIS-IV (Wechsler Adult Intelligence Scale) [49]. On all subsequent test days, a range of psychological questionnaires were administered. Chronic stress in the last month was self-reported with the Perceived Stress Scale (PSS) [50], with scores possible from 0 to 40. Furthermore, the 59-item UPPS Impulsive Behavior Scale (UPPS-P) was used to explore impulsivity [50]. The UPPS-P indicates no time reference and evaluates five dimensions of impulsivity.

#### Blood sample collection

A blood sample was collected into a BD Vacutainer^®^ K2-EDTA tube (ref. BD 367525) to obtain plasma. Venipuncture took place between 7:30–15:30 during the first study visit at the respective Departments of Psychiatry at Goethe University Hospital Frankfurt, Semmelweis University Budapest, Hungary, and Universitari Vall d’Hebron, Barcelona, Spain. Participants were fasted for at least 8 hours prior to blood sampling. After allowing blood to settle for at least 30 minutes, but no longer than 2 hours, it was centrifuged at 4 degrees Celsius and 20000 rpm for 15 min. All plasma samples were aliquoted into cryotubes and stored at -80 degrees Celsius until further analysis and unfrozen for the first time for this analysis. All procedures were completed in the period between May 2019 and March 2021.

### Biochemical analysis

Proteomic analysis was performed via Olink Target 96 Inflammation panel (Olink Proteomics, Uppsala, Sweden) [51]. Collected plasma samples were used to simultaneously detect levels of 92 inflammation-related protein biomarkers. Reagents are based on Proximity Extension Assay (PEA) technology [52]. The assay is performed in a homogeneous 96-well format. Internal controls are added to each sample, including two Immunoassay controls (extension and detection control). An external inter-plate control is included on each plate and is used in a second normalization step. Intraassay variation was 7%, and inter-assay variation (between-run) was 18%. Final levels of protein markers were recorded as normalized log2 scaled protein expression (NPX) values.

**Fig. 1:**
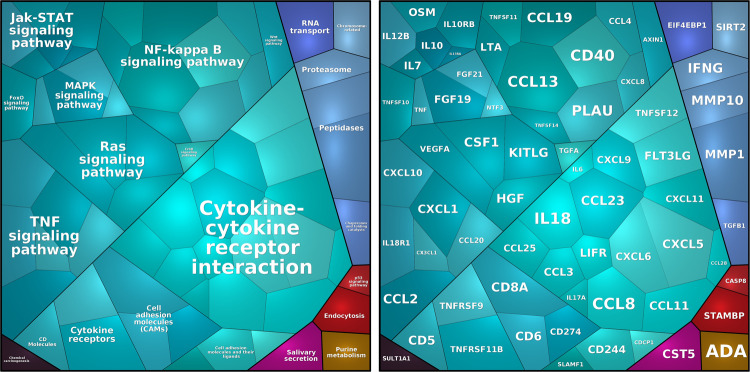
Voronoi Treemap. Involved pathways and proteins, as well as the intensity of detected inflammatory protein levels within each pathway, are derived from 126 ADHD participants from the PROBIA Study. Larger tiles indicate a more important contribution to the pathway and are based on the median values of our sample. Left panel: Involved pathways; Right panel: Involved proteins.

The assessed proteins are depicted in the Voronoi treemap [53], based on KEGG BRITE [54] nomenclature (Fig. 1). Investigated proteins were involved in Jak-STAT, NF-κB, MAPK, RAS, and TNF signaling pathway, cytokine-cytokine receptor interaction, Peptidase, cell adhesion molecules, and other processes.

### Statistical analysis

Statistical analyses were performed using R (4.1.3) [55]. A *p* value ≤ 0.05 was used as a threshold for statistical significance in the MANCOVA and as FDR-adjusted *p* value in the follow-up ANOVAS/ANCOVAs and demographic comparisons.

#### Data preprocessing and demographics

Inflammatory proteins with >50% of the samples under the protein-specific Lower Level of Detection (LLOD) were excluded as missing values. All other proteins were imputed with a random forest imputation. As indicated, descriptive statistics between groups were computed using Welch Two Sample *t* test, Wilcoxon rank sum test, Pearson’s Chi-squared test, and Fisher’s exact test. If data were not normally distributed, appropriate analyses were applied, and data were presented as median ± interquartile ranges (IQR). *P* values were adjusted for FDR with Benjamini & Hochberg correction (*p*.adj). Missing demographic data were not imputed. Power calculation for the achieved sample size (*N* = 126, Effect size = 0.25, *α*-error probability = 0.05) revealed a power of 80% (see Supplementary info).

#### Clustering and main analysis

An unsupervised cluster analysis of protein levels was performed to assess the association of inflammatory proteins with ADHD sample characteristics. Euclidian distance and the ward.2 was applied for the cluster analysis [51]. The optimal number of clusters was found based on the majority index (package NbClust [56]). Bootstrapping (package *fbc* [57, 58]) was further applied to validate participant cluster assignment using the cluster stability index based on the *Jaccard coefficient* γ [57]. Values above 0.5 are considered a stable cluster assignment. Rescaled data (mean = 0, standard deviation (sd) = 1) was visualized using the complex heatmap [59] package. Differences in imputed (but not rescaled) protein levels between patient cluster groups were then compared using a Multivariate ANOVA with the patient cluster as independent variable and study site and sex as a covariate. If the distribution was not normal, protein levels were transformed via Tukey’s Ladder of Powers (applied for IL-17A, AXIN1, SCF, Fibroblast growth factors (FGF)-23, CC-Chemokin-Ligand (CCL)-19, SIRT2, EN-RAGE, IFN-γ, Caspase 8 (CASP-8), Neurotrophin (NT)-3, CCL20, STAMBP). Homoscedasticity assumptions were checked using Levene’s test and visual inspection. If assumptions for homogeneity of variance were not met, data were transformed, and if insufficient improvement was obtained, a non-parametric test was performed to verify results. Protein clusters with a significant Pillai’s Trace Test (V) were further analyzed via a post-hoc ANOVA. Corrections for FDR were applied within each protein cluster. In addition, sample characteristics (demographical and psychological) were compared between both patient groups; if not normally distributed, data was transformed via Tukey’s Ladder of Powers (applied for age, BMI, inattention, hyperactivity, and impulsivity). Finally, post-hoc tests were conducted on the association between stress and inflammation, by calculating a composite score of Cluster 6, using Cronbach’s α of 0.7 or above to determine which proteins should be included. Only three proteins had scores below 0.7 and were not included (IL-8: *α* = 0.69, MMP1: *α* = 0.65, and CCL4: *α* = 0.66). Next, Spearman’s rank correlation (Spearman’s rho) was computed between the composite score and PSS scores, applied for the whole group and for females and males separately. Via the same principles, we also added a Spearman’s rank correlation between the composite score for Cluster 7 and suicide risk.

#### Effect of medication

The effect of medication was assessed with three different models: differences between (a) all ADHD medication-naive participants versus participants on any ADHD medication, (b) classes of ADHD medication (MPH versus LDX compared to the unmedicated group), and c) the impact of psychotropic co-medication (i.e., mood stabilizers such as atypical antipsychotic (e.g., quetiapine) and anticonvulsive medication (e.g., lamotrigine) and antidepressant medication such as Selective Serotonin Reuptake Inhibitors (SSRI, e.g., escitalopram), Selective Serotonin Noradrenalin Reuptake Inhibitors (SNRI, e.g., duloxetine), Tricyclic Antidepressants (TCA, e.g., amitriptyline) and other classes (taken only by few patients, e.g., bupropion and mirtazapine), see Supplementary Table 1 for an overview) in combination with ADHD medication (main and interaction effects of ADHD medicated versus ADHD unmedicated and psychotropic medicated versus psychotropic unmedicated). A MANOVA with imputed data was applied. Due to significant differences, the study site was used as a covariate for the first medication (a) analysis. If Pillai’s Trace Test was significant, a post-hoc ANOVA and FDR correction followed. Sensitivity analyses were also conducted excluding patients receiving immunomodulatory medication with an indication for diabetes (*N* = 2), asthma/COPD (*N* = 4), breast cancer (*N* = 1), Hashimoto (*N* = 2), cardiovascular disease (Cox-2 inhibitor, *N* = 1) and HIV (*N* = 1).

## Results

### Demographics

Of the 126 ADHD participants included in the PROBIA study, 76 participants (60,3%) received medication for ADHD (medicated; 23 received LDX, and 46 received MPH, one both types and seven other medication such as atomoxetine or guanfacine). Fifty participants received no ADHD medication (unmedicated). Co-medication for other psychiatric disorders was common: 45 (36%) subjects also took at least one type of psychotropic medication. Since the study site was significant between the groups “ADHD medicated” and “ADHD unmedicated” before FDR (*p* value = 0.006, *p*.adj = 0.067), it was included as a covariate in subsequent analyses. No additional significant differences in sample characteristics occurred between the groups “ADHD medicated” and “ADHD unmedicated” (Table 1a, b). Details on co-medication can be found in Supplementary Table 1.

**Table 1a:** Demographics of study population of ADHD participants, split by medication status.

Characteristics	*N*	Overall	Medicated	Unmedicated	*p* value (Medicated vs. unmedicated)	*p*. adj^3^
		*N* = 126	*N* = 76	*N* = 50		
Age^1a,4^	126	40 (28, 48)	42 (28, 50)	38 (11)	0.394^2a^	0.69
Sex^1b^	126				0.263^2b^	0.69
Female		73 (58%)	41 (54%)	32 (64%)		
Male		53 (42%)	35 (46%)	18 (36%)		
BMI (kg/m^2^)^1a^	125	25.2 (22.0, 28.9)	25.6 (22.5, 29.6)	25.0 (21.9, 28.0)	0.427^2a^	0.69
ADHD-diagnosis^1b^	126				0.596^2c^	0.69
Combined type		97 (77%)	60 (79%)	37 (74%)		
Predominantly inattentive type		24 (19%)	14 (18%)	10 (20%)		
Predominantly hyperactive-impulsive type		5 (4.0%)	2 (2.6%)	3 (6.0%)		
Ethnicity^1b^	126				0.312^2c^	0.69
White (Caucasian)		97 (77%)	56 (74%)	41 (82%)		
Other ethnic group		24 (19%)	16 (21%)	8 (16%)		
Asian		3 (2.4%)	3 (3.9%)	0 (0%)		
American Indian		1 (0.8%)	1 (1.3%)	0 (0%)		
Hispanic or Latino		1 (0.8%)	0 (0%)	1 (2.0%)		
Tobacco use^1b^	126				0.627^2c^	0.69
Non-smoker		66 (52%)	41 (54%)	25 (50%)		
Smoker		55 (44%)	33 (43%)	22 (44%)		
Occasional-Smokers		5 (4.0%)	2 (2.6%)	3 (6.0%)		
Vegan/vegetarian^1b^	**126**	7 (5.6%)	3 (3.9%)	4 (8.0%)	0.434^2c^	0.684
ISCE^1b^	125				0.462^2c^	0.69
Tertiary education		95 (76%)	56 (74%)	39 (80%)		
Secondary education		19 (15%)	13 (17%)	6 (12%)		
Not classified		6 (4.8%)	5 (6.6%)	1 (2.0%)		
Primary education		5 (4.0%)	2 (2.6%)	3 (6.1%)		
Salary^1b^	116				0.564^2b^	0.69
equal to mean salary of country		47 (41%)	27 (40%)	20 (41%)		
<mean salary		45 (39%)	24 (36%)	21 (43%)		
>mean salary		24 (21%)	16 (24%)	8 (16%)		
ADHD in family^1b^	126				0.739^2b^	0.739
No		51 (40%)	30 (39%)	21 (42%)		
Unknown		40 (32%)	23 (30%)	17 (34%)		
Yes		35 (28%)	23 (30%)	12 (24%)		
Psychotropic medication^1b^	126	45 (36%)	31 (41%)	14 (28%)	0.143^2b^	0.69
Study site^1b^	126				**0.006**^2b^	0.067
Barcelona		43 (34%)	34 (45%)	9 (18%)		
Frankfurt		42 (33%)	23 (30%)	19 (38%)		
Budapest		41 (33%)	19 (25%)	22 (44%)		

*ISCE*: International Standard Classification of Education. Bold values indicate significant *p* values.

^1a^Median (IQR);

^1b^*n* (%).

^2a^Wilcoxon rank sum test;

^2b^ Pearson’s Chi-squared test (% of total column);

^2c^Fisher’s exact test (% of total column).

^3^Benjamini & Hochberg correction for multiple testing.

^4^in years, estimated by the calculation of (current year−birth year).

**Table 1b:** Psychological characteristics of the overall patient group and split by medication status.

Psychological characteristics	*N*	Overall	Medicated	Unmedicated	*p* value	*p*.adj^3^
		*N* = 126	*N* = 76	*N* = 50		
ADHD rating scale^1c^	124	33 (9)	31 (9)	34 (7)	0.095^2d^	0.323
Inattention (ADHD-RS)^1a^	125	17.0 (14.0, 20.0)	16.0 (12.0, 19.5)	18.0 (15.2, 20.0)	0.117^2a^	0.323
Hyperactivity (ADHD-RS)^1a^	125	9.0 (6.0, 11.0)	9.0 (5.0, 11.0)	10.0 (6.0, 11.0)	0.489^2a^	0.672
Impulsivity (ADHD-RS)^1a^	124	7.0 (5.8, 10.0)	7.0 (5.0, 9.0)	8.0 (6.0, 10.0)	0.343^2a^	0.539
UPPS-P^1c^	113	139 (16)	139 (16)	138 (15)	0.852^2d^	0.937
Major depressive episode^1b^	126	30 (24%)	13 (17%)	17 (34%)	**0.029** ^2b^	0.162
Comorbid BPD^1b^	126	18 (14%)	6 (7.9%)	12 (24%)	**0.011** ^2b^	0.126
Suicidality risk^1b^	126				0.293^2c^	0.941
None		74 (59%)	49 (64%)	25 (50%)		
Low		44 (35%)	24 (32%)	20 (40%)		
Medium		5 (4.0%)	2 (2.6%)	3 (6.0%)		
High		3 (2.4%)	1 (1.3%)	2 (4.0%)		
CGI-S score^1b^	126				0.627^2c^	0.767
4		66 (52%)	37 (49%)	29 (58%)		
5		44 (35%)	29 (38%)	15 (30%)		
6		14 (11%)	8 (11%)	6 (12%)		
7		2 (1.6%)	2 (2.6%)	0 (0%)		
Perceived stress scale^1c^	123	22 (7)	21 (7)	23 (6)	0.336^2d^	0.539

Inattention, hyperactivity, and impulsivity as subscores from ADHD Rating Scale. UPPS: Impulsive behavior scale, Perceived Stress Scale: sum of Perceived Stress Scale (PSS). Major Depressive Episode and Suicidality risk based on M.I.N.I., Comorbid BPD based on SCID-II. CGI-S score: Score of Clinical Global Impression—severity Scale (CGI-S scores had to be four or higher for inclusion). Bold values indicate significant *p* values.

^1a^Median (IQR);

^1b^*n* (%);

^1c^Mean (SD).

^2a^Wilcoxon rank sum test;

^2b^Pearson’s Chi-squared test (% of total column);

^2c^Fisher’s exact test (% of total column);

^2d^Welch Two Sample *t* test.

^3^Benjamini & Hochberg correction for multiple testing.

### Patient biotypes with low and high inflammatory potential

**Fig. 2:**
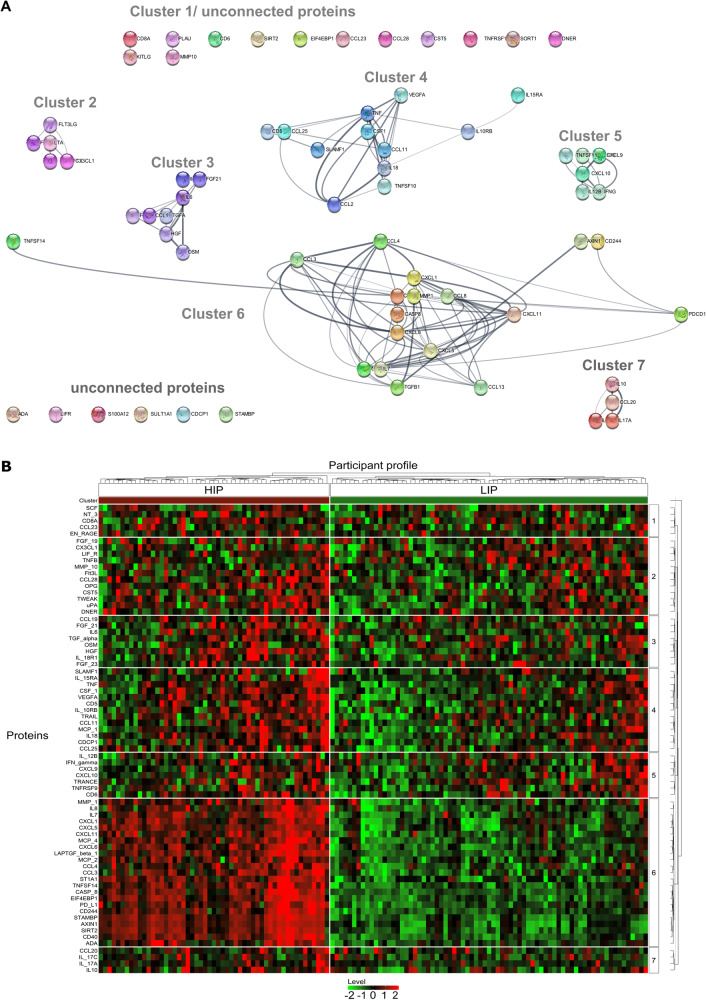
Protein and Patients Clusters. **A** Representation of the identified hierarchical protein clusters. Graph was constructed on merged networks of the predefined clusters using cytoscape [88]. **B** Heatmap of protein expression from 126 ADHD participants from the PROBIA study. Rows present proteins, while columns show each participant’s protein profile. Values are on a log scale and higher protein levels were visualized in red and lower in green. Protein levels are presented as rescaled (mean = 0, sd = 1) normalized log2 scaled protein expression (NPX) values. PDCD1 = PD-L1, CD254 = TRANCE, CD253 = TRAIL, TGFb1 = LAPTGF-β1, MCP-4 = CCL13, ST1A1 = SULT1A1, MCP-2 = CCL8, MCP-1 = CCL2.

An optimal number of two patient “biotypes” (where biotype refers to a subgroup of participants with similar levels of proteins), was identified using the majority index of the *NbClust* package [56]. Via bootstrapping, stability indices were identified as 0.88 and 0.81 (cluster-wise Jaccard bootstrap mean) for participant cluster assignments 1 and 2, respectively, indicating good stability. The cluster assignment yielded participants with relatively lower and higher inflammatory protein profiles. Hereafter, these groups will be referred to as low inflammatory potential [LIP, *N* = 73 (57,9%)] and high inflammatory potential [HIP, *N* = 53 (42,1%)] biotypes. The hierarchical cluster partition results are visualized in the heatmap (Fig. 2). Seven different protein clusters were identified. All protein clusters except for cluster 5 differed significantly between LIP and HIP groups as assessed by MANOVA. A detailed description of clusters is provided below.

#### Protein clusters

The 72 proteins were sorted into 7 different protein clusters (see Fig. 2 and Table 2): Cluster 1 differed between the biotypes and contained chemokines involved in (T-cell) immune responses (*V* = 0.10, *F* = 2.50, *p* value = 0.034). After correction for FDR, only one of the five proteins remained significant (NTF-3; post-hoc ANOVA: *p* value = 0.003, *p*.adj = 0.014). Cluster 2 contained 12 chemoattraction and cell differentiation proteins and differed significantly between HIP and LIP (*V* = 0.29, *F* = 3.69, *p* value < 0.001). Four proteins were still significant between the LIP and HIP groups after FDR correction (CCL28, TWEAK, Urokinase (uPA), and Osteoprotegerin (OPG); post-hoc ANOVA: *p* value ≤ 0.006, *p*.adj ≤0.017, see Table 2 for complete values). Cluster 3 and 4 both contained classical pro-inflammatory proteins and proteins involved in immune metabolism, both significantly different between groups (Cluster 3: *V* = 0.34, *F* = 7.33, *p* value < 0.001; Cluster 4: *V* = 0.33, *F* = 4.02, *p* value < 0.001). Protein cluster 3 contained eight proteins, and cluster 4 thirteen proteins, of which three and ten proteins (see Table 2) were significant after FDR, respectively. Cluster 5 contained predominantly proteins related to T-cell immune responses and was not significantly different between groups (*V* = 0.10, *F* = 1.82, *p* value = 0.089). Cluster 6 was the largest cluster, containing several proteins involved in the NF-κB pathway and prostaglandin activation. It showed the strongest difference between both groups (*V* = 0.87, *F* = 29.64, *p* value < 0.001), with all 23 proteins remaining significantly different after FDR. Finally, the small Cluster 7 contained proteins related to the IL-17 signaling cascade and was significantly different between both groups (*V* = 0.11, *F* = 3.55, *p* value = 0.009), with 3 out of 4 (IL-17A, IL-17C, and IL-10) significantly different between the groups after FDR. Table 2 summarizes all proteins and differences between groups, and Supplementary Table 2 describes protein functions within the clusters. Additional analyses excluding all patients with potential immunomodulatory medication, did not alter the significance or direction of the results, and the model, including sex as a factor, showed a significant effect of sex for most clusters, but this did not alter the significance for estimates between HIP and LIP groups (Supplementary Table 3).

**Table 2:** Differences in proteins between HIP and LIP based on assigned cluster.

Cluster	Protein	LIP		HIP		HIP vs. LIP		
		(*N* = 73)		(*N* = 53)				
		mean	sd	mean	sd	F^1^	*p* value^1^	*p*.adj^2^
Cluster 1	EN-RAGE	2.14	0.60	2.31	0.69	2.78	0.098	0.245
	CCL23	9.66	0.47	9.73	0.44	0.81	0.368	0.461
	CD8A	9.45	0.62	9.59	0.67	1.31	0.255	0.425
	NT-3	1.88	0.36	2.10	0.48	9.30	**0.003**	**0.014**
	SCF	9.11	0.34	9.08	0.36	0.17	0.682	0.682
Cluster 2	DNER	8.40	0.26	8.43	0.23	0.50	0.481	0.578
	uPA	9.38	0.22	9.59	0.26	25.55	**<0.001**	**<0.001**
	TWEAK	8.51	0.26	8.64	0.27	7.92	**0.006**	**0.017**
	CST5	5.99	0.43	6.15	0.46	4.01	**0.048**	0.111
	OPG	9.64	0.28	9.85	0.30	16.47	**<0.001**	**0.001**
	CCL28	2.19	0.32	2.38	0.39	9.33	**0.003**	**0.011**
	MMP-10	8.98	0.74	8.94	0.72	0.09	0.758	0.827
	TNFB	4.61	0.40	4.54	0.34	1.06	0.305	0.523
	LIF-R	3.72	0.21	3.75	0.22	0.57	0.45	0.578
	Flt3L	8.59	0.39	8.72	0.41	3.75	0.055	0.111
	CX3CL1	3.82	0.35	3.88	0.37	0.82	0.366	0.549
	FGF-19	7.75	0.83	7.74	0.95	<0.01	0.955	0.955
Cluster 3	FGF-23	1.84	0.30	1.91	0.31	2.54	0.114	0.152
	IL-18R1	7.71	0.37	7.86	0.47	4.57	**0.035**	0.069
	HGF	7.68	0.35	8.09	0.43	36.93	**<0.001**	**<0.001**
	OSM	4.18	0.80	4.08	0.88	0.51	0.475	0.475
	TGF-α	2.41	0.25	2.46	0.23	1.42	0.236	0.27
	IL6	1.70	0.49	1.87	0.52	3.33	0.071	0.113
	FGF-21	4.54	1.13	5.08	1.42	5.73	**0.018**	**0.048**
	CCL19	8.59	0.62	8.87	0.70	7.73	**0.006**	**0.025**
Cluster 4	CCL25	5.62	0.46	5.92	0.48	12.92	**<0.001**	**0.001**
	CDCP1	2.04	0.48	2.33	0.69	7.99	**0.006**	**0.007**
	IL18	8.32	0.52	8.75	0.55	19.85	**<0.001**	**<0.001**
	MCP-1	10.48	0.36	10.68	0.35	8.99	**0.003**	**0.005**
	CCL11	7.22	0.41	7.35	0.50	9.55	0.095	0.095
	TRAIL	7.52	0.30	7.67	0.27	8.15	**0.005**	**0.007**
	IL-10RB	5.74	0.26	5.94	0.26	19.20	**<0.001**	**<0.001**
	CD5	4.94	0.27	5.10	0.28	10.59	**0.001**	**0.003**
	VEGFA	10.41	0.29	10.77	0.36	38.99	**<0.001**	**<0.001**
	CSF-1	9.68	0.19	9.80	0.19	11.00	**0.001**	**0.003**
	TNF	2.05	0.33	2.29	0.30	17.67	**<0.001**	**<0.001**
	IL-15RA	0.94	0.08	0.98	0.16	3.52	0.063	0.068
	SLAMF1	2.10	0.30	2.22	0.40	3.87	0.052	0.061
Cluster 5	CD6	5.88	0.42	6.06	0.38	-	-	-
	TNFRSF9	6.21	0.35	6.34	0.35	-	-	-
	TRANCE	4.47	0.63	4.50	0.65	-	-	-
	CXCL10	8.94	0.80	9.20	0.71	-	-	-
	CXCL9	6.04	0.66	6.13	0.50	-	-	-
	IFN-γ	6.23	0.80	6.36	0.81	-	-	-
	IL-12B	5.85	0.56	5.79	0.60	-	-	-
Cluster 6	ADA	5.04	0.32	5.68	0.62	72.53	**<0001**	**<0.001**
	CD40	10.46	0.37	11.67	0.49	344.60	**<0.001**	**<0.001**
	SIRT2	3.95	1.08	7.29	1.13	387.69	**<0.001**	**<0.001**
	AXIN1*	3.68	1.70	7.58	1.10	514.12	**<0.001**	**<0.001**
	STAMBP	4.26	0.97	7.53	1.04	622.81	**<0.001**	**<0.001**
	CD244	5.66	0.25	6.43	0.43	185.77	**<0.001**	**<0.001**
	PD-L1	4.96	0.24	5.61	0.38	149.54	**<0.001**	**<0.001**
	EIF4EBP1	7.51	0.70	9.73	0.83	369.42	**<0.001**	**<0.001**
	CASP8	1.71	0.28	2.76	0.59	223.47	**<0.001**	**<0.001**
	TNFSF14	3.52	0.49	4.69	0.54	167.40	**<0.001**	**<0.001**
	ST1A1*	2.53	0.61	3.73	0.56	143.19	**<0.001**	**<0.001**
	CCL3	4.61	0.36	5.23	0.43	78.04	**<0.001**	**<0.001**
	CCL4	5.13	0.47	5.81	0.51	59.69	**<0.001**	**<0.001**
	MCP-2	7.84	0.58	8.67	0.63	59.18	**<0.001**	**<0.001**
	LAPTGF β-1	5.34	0.28	6.32	0.49	206.91	**<0.001**	**<0.001**
	CXCL6	7.40	0.68	9.03	0.78	190.25	**<0.001**	**<0.001**
	MCP-4	12.87	0.60	14.21	0.71	128.95	**<0.001**	**<0.001**
	CXCL11	6.58	0.70	7.91	0.77	110.46	**<0.001**	**<0.001**
	CXCL5	9.80	1.41	12.24	0.77	146.55	**<0.001**	**<0.001**
	CXCL1	8.92	0.93	10.49	0.76	116.35	**<0.001**	**<0.001**
	IL-8	3.99	0.45	4.69	0.51	67.17	**<0.001**	**<0.001**
	IL-7	1.71	0.51	2.76	0.58	152.07	**<0.001**	**<0.001**
	MMP1	12.70	0.99	13.92	0.89	52.15	**<0.001**	**<0.001**
Cluster 7	IL-17A	1.69	0.27	1.89	0.55	8.52	**0.004**	**0.013**
	IL-17C	2.74	0.66	3.08	1.05	4.88	**0.029**	**0.039**
	IL-10	3.14	0.41	3.35	0.43	7.67	**0.007**	**0.013**
	CCL20	6.91	0.86	7.20	1.07	3.63	0.059	0.059

Protein levels are presented as untransformed, normalized log2 scaled protein expression (NPX) values. Cluster 6 shows the highest difference between high and low inflammatory potential groups. Bold values indicate significant *p* values.

^1^Post-hoc ANOVA.

^2^Benjamini & Hochberg correction for multiple testing within cluster.

*Due to significant Levene’s test, the results for these proteins were also verified with a non-parametric test, with similar results.

#### Demographic and psychological differences between HIP and LIP

Next, patient clinical characteristics were explored between the HIP/LIP groups. Participants in the HIP group presented higher stress levels (*p* value < 0.001) together with higher CGI-S scores (*p* value = 0.030) and more moderate to higher ratings for suicide risk (*p* value = 0.032, see Table 3). Given the recent interest in suicidality and IL-17 signaling, we additionally explored whether cluster 7 was associated with the different suicide risk classes but found no difference between groups (see Supplementary Fig. 1). There was also no association between HIP/LIP status and ADHD Rating Scale Scores (*p* value = 0.531) or increased subscale scores (*p* value ≥ 0.153, see Table 3). Regarding other demographics, the two groups differed significantly between sites (*p* value < 0.001). Most participants from Frankfurt were assigned to the HIP group, while the biggest part of participants from Budapest and Barcelona were in the LIP group. Moreover, the HIP group was associated with higher psychotropic medication intake (*p* value = 0.002). Of note, no association with ADHD medication nor other demographics such as sex, BMI, obesity, tobacco use, vegan/vegetarian nutrition, or age was found between LIP and HIP groups. (see Supplementary Table 4a). Further investigation of macro- and micronutrients did not reveal major differences between LIP and HIP groups, including inflammation-associated nutrients such as sodium and fat or those with anti-inflammatory properties. Yet, given the heterogeneous assessment methods, we were only able to compare each site separately for HIP and LIP groups (Supplementary Table 4b). Numerically, particularly in the Frankfurt group, omega 3 and docosahexaenoic acid consumption was slightly higher in the LIP group, but this was not true for the Barcelona group. In the Barcelona group, omega 6 fatty acids were higher in the HIP group.

**Table 3:** Psychological characteristics of HIP and LIP.

Psychological	*N*	HIP	LIP	*p* value	*p*.adj^3^
Characteristics		*N* = 53	*N* = 73		
ADHD rating scale^1c^	124	33 (8)	32 (9)	0.531^2d^	0.664
Inattention (ADHD-RS)^1c^	125	70 (25)	60 (29)	0.153^2d^	0.307
Hyperactivity (ADHD-RS)^1a^	125	17 (9, 19)	15 (7, 17)	0.964^2a^	0.964
Impulsivity (ADHD-RS)^1a^	124	9.4 (6.9, 13.2)	8.1 (5.6, 10.6)	0.770^2a^	0.855
Major depressive episode^1b^	126	15 (28%)	15 (21%)	0.313^2b^	0.522
Comorbid BPD^1b^	126	11 (21%)	7 (9.6%)	0.077^2b^	0.193
Suicidality risk^1b^	126			**0.032**^2c^	0.106
None		29 (55%)	45 (62%)		
Low		17 (32%)	27 (37%)		
Medium		5 (9.4%)	0 (0%)		
High		2 (3.8%)	1 (1.4%)		
CGI-S score^1b^	126			**0.030**^2c^	0.106
4		23 (43%)	43 (59%)		
5		18 (34%)	26 (36%)		
6		10 (19%)	4 (14%)		
7		2 (3.8%)	0 (0%)		
Perceived stress scale^1c^	123	24 (7)	20 (6)	**<0.001**^2d^	**0.004**

Inattention, hyperactivity, and impulsivity as subscores from ADHD Rating Scale (ADHD-RS) and transformed with Tukey’s ladder of powers. Major depressive episode and suicidality risk based on M.I.N.I., Comorbid BPD based on SCID-II. Perceived stress scale: sum of Perceived Stress Scale (PSS). CGI-S score: Score of Clinical Global Impression—severity scale (CGI-S scores had to be four or higher for inclusion). Bold values indicate significant *p* values.

^1a^ Median (IQR);

^1b^*n* (%);

^1c^Mean (SD).

^2a^Wilcoxon rank sum test;

^2*b*^Pearson’s Chi-squared test (% of total column);

^2c^Fisher’s exact test (% of total column);

^2d^Welch Two Sample *t* test.

^3^Benjamini & Hochberg correction for multiple testing.

### Inflammatory proteins are associated with perceived chronic stress levels

**Fig. 3:**
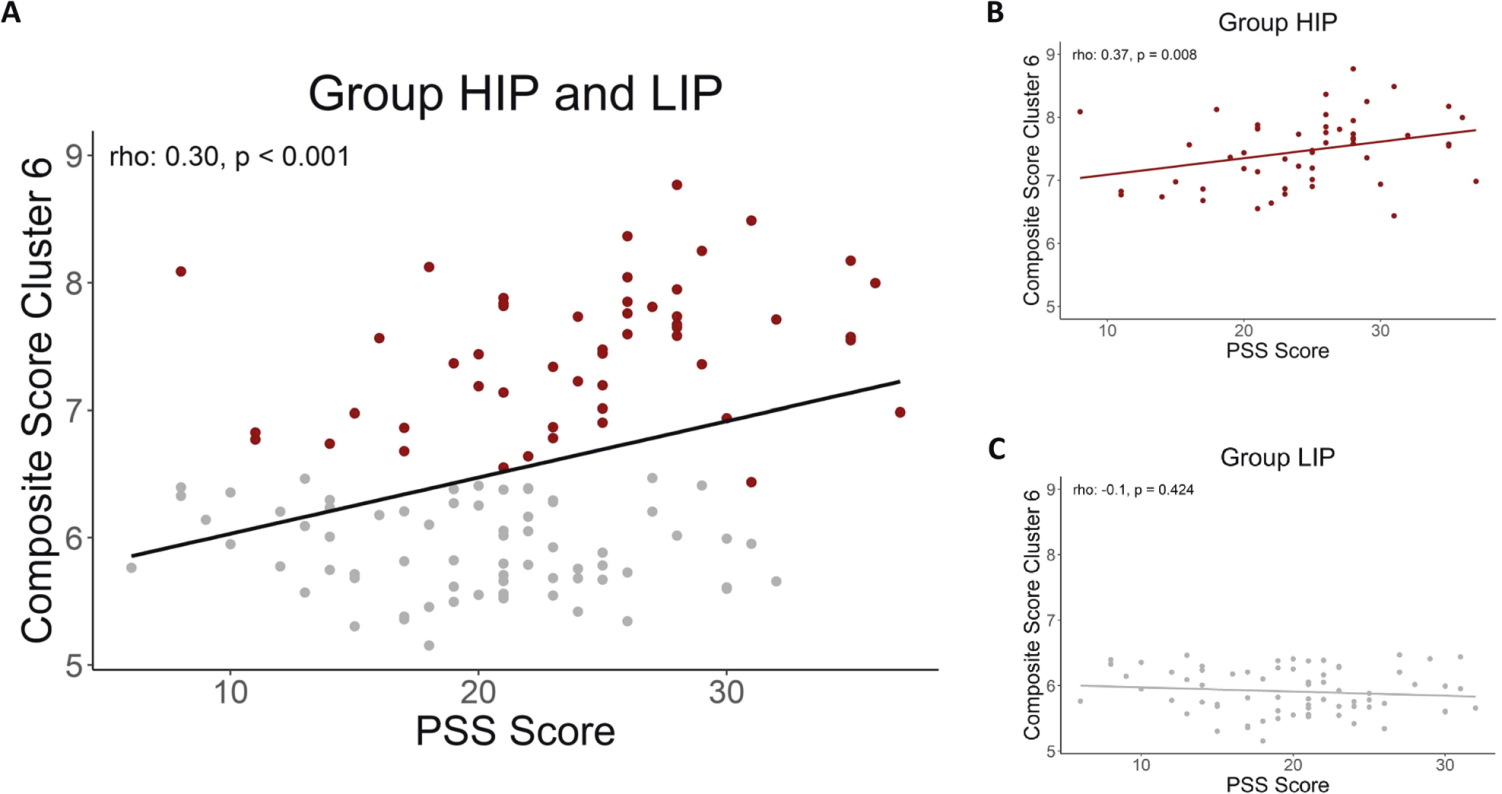
Correlation of Protein Cluster 6 and chronic stress ratings. **A** Association between the composite score and chronic stress levels (PSS Score) in all aADHD participants. Gray dots indicate people in the LIP group, whereas red dots indicate people in the HIP group. **B** Correlation only for HIP group. **C** Correlation only for LIP group.

Chronic stress was significantly associated with inflammatory protein expression in cluster 6 (Spearman’s rho = 0.30, df = 124, *p* value < 0.001, see Fig. 3). No significant correlation was observed between perceived stress and the composite score in the LIP group (Spearman’s rho = −0.1, df = 124, *p* value = 0.424). In contrast, a substantial positive correlation was observed in the HIP group (Spearman’s rho = 0.37, df = 124, *p* value = 0.008). Furthermore, we investigated whether sex differences were present. We did not find any difference in males and females: the overall correlation between PSS and composite cluster 6 was positive and significant for both females (Spearman’s rho = 0.32, *p* value = 0.007), and males (Spearman’s rho = 0.34, *p* value = 0.013). Splitting further for HIP/LIP clusters showed similar patterns as the main analysis, albeit significance was reduced to a trend (Females: HIP Spearman’s rho = 0.35, *p* = 0.057; LIP Spearman’s rho = 0.09, *p* = 0.567; Males: HIP Spearman’s rho = 0.37, *p* value = 0.095, LIP Spearman’s rho = −0.18, *p* value = 0.309, see Supplementary Fig. 2).

### Inflammatory protein levels and ADHD medication status

We further explored whether group differences between medicated and unmedicated participants with ADHD were present. No difference between ADHD-specific-medicated and unmedicated participants with ADHD was observed (Cluster 1: *p* value = 0.197, Cluster 2: *p* value = 0.592, Cluster 3: *p* value = 0.074, Cluster 4: *p* value = 0.502, Cluster 5: *p* value = 0.182, Cluster 6: *p* value = 0.126, Cluster 7: *p* value = 0.108). ADHD severity also did not differ between medicated and unmedicated groups (see Table 1b). The comparison between people receiving MPH vs. LDX and unmedicated participants with ADHD revealed significant differences in the MANOVA for Cluster 6 (*V* = 0.53, *F* = 1.50, *p* value = 0.031). However, no single protein was significantly different between groups in the post-hoc ANOVA analysis (see Supplementary Table 5). Finally, psychotropic medication influenced protein levels significantly for Cluster 2, and 4 (Cluster 2: *V* = 0.20, *F* = 2.37, *p* value = 0.009, Cluster 4: *V* = 0.27, *F* = 3.19, *p* value < 0.001). Follow-up analyses revealed significantly higher levels of FLT3, CCL25, CDCP1, IL-18, CCL11, IL-10RB, VEGFA, and IL-15RA in those receiving psychotropic medication compared to unmedicated participants (all *p* values < 0.05). A detailed overview of post-hoc ANOVA results can be found in Supplementary Table 6.

## Discussion

In the present study, a data-driven approach was used to explore plasma proteomic signatures of inflammation in one of the largest available datasets of adults with ADHD. We found evidence for a heterogeneous distribution of inflammatory proteins and the presence of two biotypes, discovered using a non-forced clustering approach. One biotype with overall lower inflammatory status (LIP), and the other with higher protein levels (HIP). The distinction was most evident in proteins related to chemokine signaling and the NF-κB pathway. The HIP group could be linked to higher levels of perceived stress, modestly higher risk for suicidality, and slightly more severe impairment based on the CGI-S. No association between inflammation and ADHD subtypes could be detected.

Protein cluster 6 showed the clearest distinction in protein levels between the two biotypes. The majority of the 23 proteins in this cluster contributed to chemokine signaling (e.g., CCL3, CCL4, CCL8, CCL13, CXCL1, CXCL5, IL-8, and CXCL11) or (indirectly) to the NF-κB pathway (e.g., CD40, IL-7, IL-8, AXIN, and TNFSF14). The chemokine signaling pathway has pleiotropic functions related to inflammation in that it leads to the recruitment and activation of immune cells and the attraction of immune cells to various tissues. The NF-κB pathway also influences innate and adaptive immune processes and can play a role in inflammasome activation [60]. It regulates gene expression involved in the immune response and triggers the production of inflammatory cytokines, chemokines, and other immune-related molecules [61]. While a (moderately) active NF-κB pathway can be linked to neuroprotection, over-activity can increase inflammatory activity [62]. Proteins of both pathways have rarely been investigated in ADHD. Two recent reports did not find IL-8 to be increased in children with ADHD [63, 64], but others found a higher likelihood between attention problems and IL-8 [65]. To our knowledge, none of the other proteins in this cluster has been linked to ADHD before. The NF-κB pathway has, however, been associated with other severe psychiatric disorders such as schizophrenia [66] and is activated in response to stress exposure [67]. It may thus present an interesting therapeutic target for stress-related disorders if confirmed in future research.

Interestingly, two distinct inflammatory biotypes were identified based on the protein levels, which may, in part, explain the heterogeneity and small effect sizes of previous findings regarding inflammation in ADHD [19, 68]. In line with the above-mentioned association between increased NF-kB signaling and stress, the most distinctive feature among participants with ADHD in the HIP and LIP groups was indeed chronic stress. The HIP group’s higher chronic stress levels also align with research in other healthy and psychiatric populations, where high perceived stress was associated with elevated inflammation markers [69, 70]. It is well established that exposure to acute stress leads to an (adaptive) increase in acute phase proteins and cytokines [36, 71]. In contrast, chronic stress may lead to premature aging of the immune system and lead to (maladaptive) chronic low-grade inflammation (i.e., “inflammaging”) [72]. It is thus possible that (some) inflammatory proteins present a more general marker for high perceived stress rather than being specific to ADHD pathology.

From a mechanistic perspective, chronic stress can disrupt HPA axis functioning by altering cortisol levels, inducing glucocorticoid resistance, and thereby disrupting an important negative feedback loop to clear inflammation [73]. Indeed, it was shown that higher perceived stress and inflammation could be linked to flatter diurnal cortisol slopes in a national study of adults [74]. In ADHD, high perceived stress is common (e.g., Combs and colleagues [75]), and a recent meta-analysis reported lower basal and diurnal cortisol levels but no change or even *lower* cytokine levels in youth with ADHD [76]. Another meta-analysis did not find differences in cortisol levels in adults with ADHD [77]. Regarding cytokines, a trend for modestly elevated cytokines was detected in the most recent meta-analysis [19], which could support the low-grade chronic inflammation hypothesis for ADHD. However, as can be seen clearly from our results, inflammatory proteins are not distributed homogeneously in ADHD: several participants in the LIP group, who were characterized by low inflammatory status, also experienced high levels of chronic stress. Our findings may point towards a biologically more resilient biotype, which is not as affected by high chronic stress levels.

It is also possible that in the LIP group, the chronic stress exposure has not (yet) led to cortisol resistance, and the relatively lower inflammatory protein levels in the light of stress are indicative of a normally functioning negative feedback loop by the HPA axis. Alternatively, people in the LIP group may rate stressful life events as more stressful, which may skew the picture. In support of this hypothesis, the CGI scores were higher in the HIP than in the LIP group. Given that the CGI represents the global clinically determined impression of illness severity, taking all factors into account, and that all patients had to be at least moderately ill to be included in the study, this finding is not negligible, indicating that inflammation was more likely to be present in more severe cases when all factors are considered, and not necessarily related to ADHD-specific symptomatology. If replicated, this finding has interesting implications for the treatment of patients with ADHD and inflammation in the light of precision medicine: stress reduction techniques or interventions targeted to reduce inflammation (i.e., exercise therapy, dietary interventions, and possibly, for cases with confirmed chronic low-grade inflammation, anti-inflammatory medication) could be essential for this patient group. It should be emphasized that at this stage, there is yet too little evidence to give treatment recommendations, and thorough replication studies are needed to move forward.

Next to chronic stress levels, adults in the HIP had a higher risk for medium or high suicide risk. They were also clinically more severely affected, as measured by the CGI-S. However, ADHD severity on the ADHD Rating Scale and comorbid depression or BPD did not differ between both groups. This is an important finding, given that comorbidity of uni- and bipolar depression in adult ADHD are very common [30, 78], and both disorders have been associated with altered inflammatory parameters. In our sample, possible inflammatory alterations can thus not be attributed to the presence of current depression, although an effect of antidepressant co-medication cannot be excluded (also see below). A wealth of literature also links suicidality with inflammation (e.g., [79]). A recent large-scale study [80] using Mendelian randomization study found the upregulation of IL-6 signaling to be associated with suicidality. The relationship between higher percentages of people with moderate or high suicidality in our HIP group partially aligns with these findings, although only a trend of higher IL-6 was observed in the HIP group, and no specific analyses were conducted for suicide risk outside of the HIP/LIP groups. Recently, in a large-scale study [81], the link between suicidality and ADHD was partially explained by perceived stress pointing towards (some) shared mechanisms. Yet, given that the numbers of people in the medium and high suicide-risk groups were very low, these results should be interpreted cautiously. Intriguingly, we have recently found that only depressed patients with a high risk for suicide [82] but not those with lower risk had elevated levels of Th17 cells. While we did not find an association between the different suicide risk classes and cluster 7 (containing proteins related to IL-17 signaling), we only had very few cases in the high-risk groups. Further investigations of the link between Th17 cells/IL-17 signaling and suicide across disorders rather than in a disease-specific context are warranted. It should also be noted that the HIP cluster had most patients from Frankfurt. While we used identical protocols, lab procedures, and usables, it is possible that local differences in nutrition/lifestyle and/or patient characteristics that we did not assess contribute to the inflammatory difference. The different composition of macro and micronutrients is indeed indicative of such effects and should be accounted for with validated methods. Against our hypothesis, our findings show no substantial link between protein levels and ADHD subtypes and/or symptoms, nor for ADHD medication status (medicated or unmedicated) or type (MPH and LDX). Regarding ADHD medication, although no comparable protein panel was assessed in other studies, Yang et al. [25] also did not find an effect of ADHD-specific medication and ADHD inflammatory proteins in their adult population, although sICAM-1 and sVCAM-1a did differ in their child population, and no effect of other proteins was found in their second publication [28]. Indeed, the previous reports on the effect of medication on inflammatory markers were limited to children and found lower levels of IFN-γ and IL-13 [20] but higher levels of IL-6 in unmedicated children [19]. However, results in the children were not controlled for stress levels, and future research is needed on this. Regarding ADHD symptomatology, previous studies have investigated the link between ADHD symptoms and inflammation in children and youth and found inconsistent results. Our results assess a far larger panel of proteins and suggest that no association between symptoms and inflammation can be found in adults.

In the past decade, the awareness of the role of dietary factors for mental health and inflammation has also increased largely. While a western diet rich in fat, salt, and sugar is known to be associated with an inflammatory diet (e.g., [83]), we found no major differences between the LIP and HIP groups, although a statistical comparison of the total group was not possible. On a numerical level, docosahexaenoic acid and omega 3 fatty acid consumption were higher in the LIP group, congruent with the anti-inflammatory effect attributed to these nutrients [84]. Omega 6 consumption was higher in the HIP group in Barcelona and omega 6 fatty acids have been described as pro-inflammatory, although there is controversy on this effect, and anti-inflammatory properties have also been described [85]. It should also be noted that the effects on inflammation are dependent on the dose, and most consumed nutrients were within the recommended doses, or below recommended doses. Furthermore, the micro- and macronutrients can only be as good as the reported data and may contain some imprecision due to omitted food items. Comparisons in larger groups with similar assessment methods are vital to assess the relationship between nutrition and inflammation. Lastly, we did find that psychotropic medication was associated with several proteins, amongst which IL-15, IL-18, and IL-10RB, which is in line with previous reports on the effect of psychotropic medication (e.g., both increasing and decreasing effects have been reported [86]). These results should, however, be interpreted with caution: only 14 people of those who did not take ADHD-specific medication (28%) received psychotropic medication, whereas 31 participants (41%) of the ADHD-medicated group did. Larger group comparisons should be conducted to ascertain this effect.

## Limitations

The present study had a number of limitations. First, in the study protocol, due to its prospective clinical trial design, no suitable control group was included. Although our analyses reveal two clearly distinct patient biotypes, we cannot situate the findings regarding people without ADHD. Next, using OLINK technology, we did not obtain concentrations of the proteins, making it difficult to compare our results to existing studies [87]. Furthermore, significant differences were observed for patients recruited at different sites despite an identical protocol and the use of identical blood collection tubes, devices, and protocols. It is possible that chronic stress levels, which were lowest in Barcelona and highest in the Frankfurt group, or other factors can influence inflammation levels. Amongst these factors are nutrition and lifestyle factors, which should be taken into account for future analyses. Lastly, this study did not assess primary proteins of the NF-kB pathway (e.g., NFkB1, NFkB2, RelA/NF-kB p65, RelB, c-Rel) but may be an interesting target for future research.

## Conclusion

In conclusion, we here presented one of the largest, well-phenotyped cohorts of adult participants with aADHD and the inflammatory proteome. We were able to identify two biotypes with distinct inflammatory profiles. The inflammatory potential group was characterized by high perceived chronic stress, a more severe clinical global impression rating, and higher suicidality risk. In this adult cohort, we did not find evidence for a cross-sectional association between ADHD medication and various cytokine levels, nor could we confirm an association with ADHD symptoms. Future studies should assess chemokines and proteins involved in the NF-kB pathway and account for chronic stress levels.

### Supplementary information

Supplementary Material

## Data Availability

The data for proteins (NPX values) and corresponding cluster attribution is available at https://gude.uni-frankfurt.de/handle/gude/323, 10.25716/gude.0cha-dnxp.

## Code availability

R code is available upon request by contacting the corresponding author.
