# Supplementary material for: Inflammatory biotype of ADHD is linked to chronic stress: a data-driven analysis of the inflammatory proteome
Source: Transl Psychiatry. 2024 Jan 18;14:37. doi: 10.1038/s41398-023-02729-3 (PMC10796401; doi:10.1038/s41398-023-02729-3)
Supplement: Supplementary file 1 — Supplementary Material [file 41398_2023_2729_MOESM1_ESM.docx]

**SUPPLEMENTARY MATERIAL**

**Supplementary Figure 1.** Boxplot of suicidaility risk (None, Low, Medium, High) and Composite Score of cluster 7.


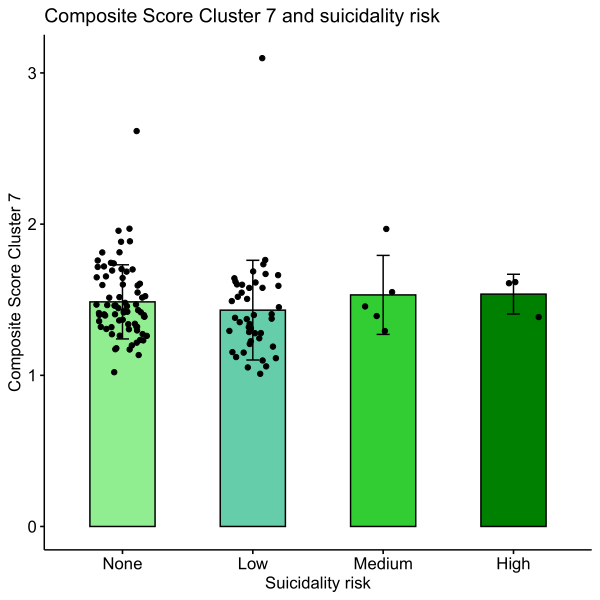


**Supplementary Figure 2. A.** Association between the composite score and chronic stress levels (PSS Score) in females from the HIP group. **B.** Association between the composite score and chronic stress levels (PSS Score) in males from the HIP group. **C.** Association between the composite score and chronic stress levels (PSS Score) in females from the LIP group **D.** Association between the composite score and chronic stress levels (PSS Score) in males form the LIP group.


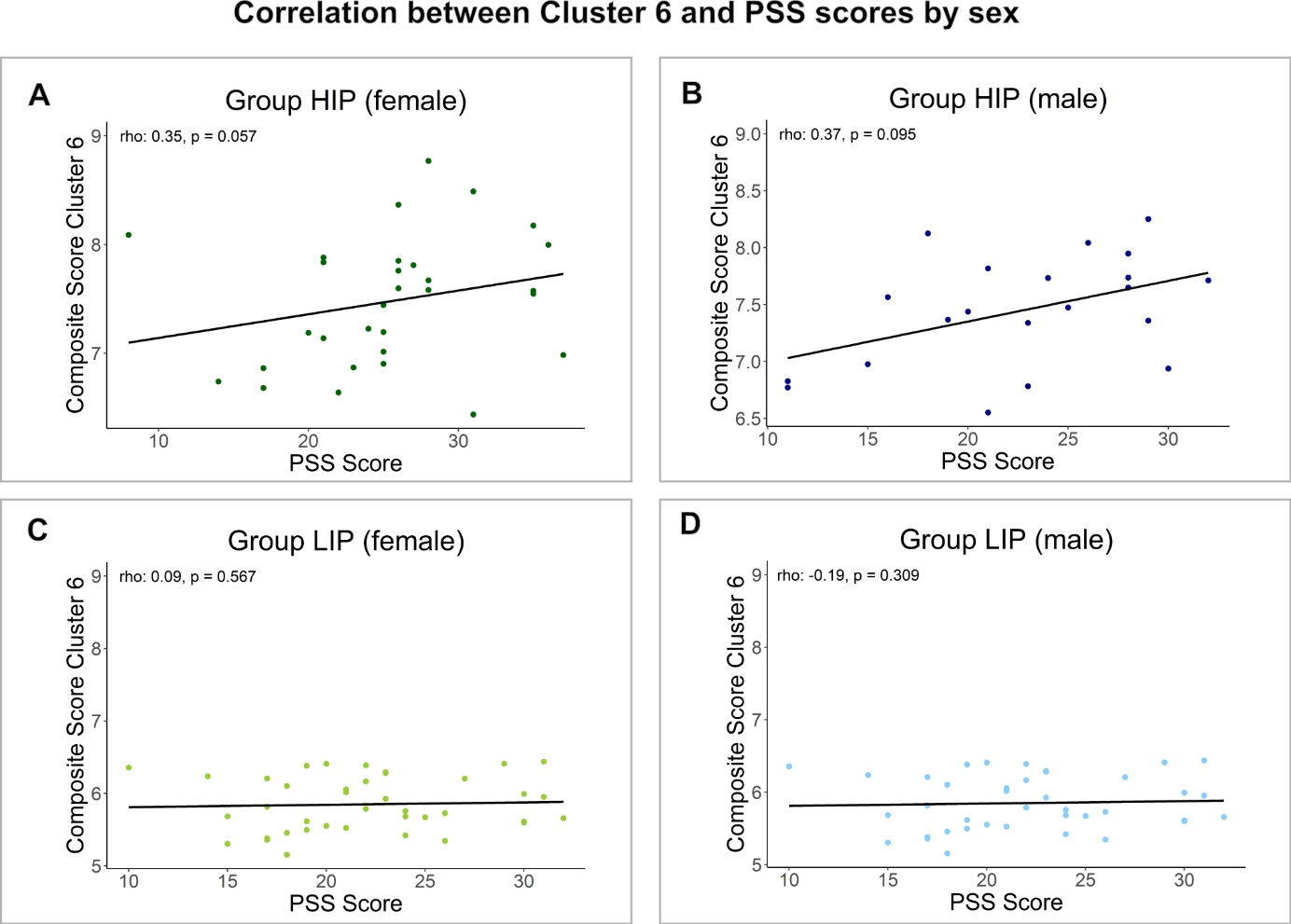


| **Supplement Table 1.** **ADHD participants’ co-medication including psychotropic medication.** Multiple data entries per participant possible. Anti-Depressant Medication and Mood Stabilization form the group “Psychotropic medication”. SSRI = Selective Serotonin Reuptake Inhibitors, SSNRI = Selective Ser-otonin Noradrenalin Reuptake Inhibitors, TCA = Tricyclic antidepressants. | |
| --- | --- |
| **Co-Medication Type** | **N** |
| **Anti-Depressant Medication** |  |
| ·        SSRI/ SNRI : escitalopram, sertraline, fluoxetine, fluvoxamine, venlafaxine, paroxetine, duloxetine | 28 |
| ·        TCA: amitriptyline, clomipramine | 2 |
| ·        Others: bupropion, agomelatine, trazodone, mirtazapine | 8 |
| **Mood Stabilization** | 12 |
| (Atypical Psychotic: quetiapine, Anticonvulsant: lamotrigine) |  |
| **Heart medication** | 15 |
| (Beta Blocker: bisoprolol, nebivolol, Calcium Antagonist: amlodipin, ACE inhibitors: enalapril, ramipril, perindopril; ASS, candesartan, telmisartan) |  |
| **Thyroid Supplement** | 11 |
| (L-Thyroxin) |  |
| **Sedative hyponotic drugs** |  |
| ·        Benzodiazepines: clonazepam, lorazepam | 9 |
| ·        Non benzodiazepines: zolpidem, zopiclone | 2 |
| **Pain Medication** | 6 |
| (triptane, gabapentine, pregabaline, tillidin, diamorphin) |  |
| **Contraceptives** | 5 |

| **Supplementary Table 2.** Protein functions within the clusters including a STRING database description and detailed information on involved cells and cell processes. Stringdb = STRING database. The description is imported from Cytoscape. | | | | | | | |
| --- | --- | --- | --- | --- | --- | --- | --- |
| **Display name** | **Query term** | **stringdb::canonical name** | **stringdb::database identifier** | **full name** | **String description** | **Cluster** | **Involved cells/processes** |
| CCL23 | CCL23 |  | 9606.ENSP00000481357 | C-C motif chemokine ligand 23 | C-C motif chemokine ligand 23. Wikipedia: CCL23 is highly chemotactic for resting T cells and monocytes and slightly chemotactic for neutrophils. It has also been attributed to an inhibitory activity on hematopoietic progenitor cells. | 1 | T cells, neutrophils, chemotaxis |
| CD8A | CD8A | P01732 | 9606.ENSP00000386559 | T-lymphocyte differentiation antigen T8/Leu-2 | T-lymphocyte differentiation antigen T8/Leu-2; Integral membrane glycoprotein that plays an essential role in the immune response and serves multiple functions in responses against both external and internal offenses. In T-cells functions primarily as a coreceptor for MHC class I molecule:peptide complex. The antigens presented by class I peptides are derived from cytosolic proteins while class II derived from extracellular proteins. Interacts simultaneously with the T-cell receptor (TCR) and the MHC class I proteins presented by antigen presenting cells (APCs). In turn recruits the Src kinase LCK to the vicinity of the TCR-CD3 complex. LCK then initiates different intracellular signaling pathways by phosphorylating various substrates ultimately leading to lymphokine production motility adhesion and activation of cytotoxic T-lymphocytes (CTLs). This mechanism enables CTLs to recognize and eliminate infected cells and tumor cells. In NK- cells the presence of CD8A homodimers at the cell surface provides a survival mechanism allowing conjugation and lysis of multiple target cells. CD8A homodimer molecules also promote the survival and differentiation of activated lymphocytes into memory CD8 T-cells. |  | T cell differentiation; NK cells, MHC I co-receptor; memory T cell generation |
| KITLG | SCF | P21583 | 9606.ENSP00000228280 | Mast cell growth factor | Mast cell growth factor; Ligand for the receptor-type protein-tyrosine kinase KIT. Plays an essential role in the regulation of cell survival and proliferation hematopoiesis stem cell maintenance gametogenesis mast cell development migration and function and in melanogenesis. KITLG/SCF binding can activate several signaling pathways. Promotes phosphorylation of PIK3R1 the regulatory subunit of phosphatidylinositol 3-kinase and subsequent activation of the kinase AKT1. KITLG/SCF and KIT also transmit signals via GRB2 and activation of RAS RAF1 and the MAP kinases MAPK1/ERK2 and/or MAPK3/ERK1. KITLG/SCF and KIT promote activation of STAT family members STAT1 STAT3 and STAT5. KITLG/SCF and KIT promote activation of PLCG1 leading to the production of the cellular signaling molecules diacylglycerol and inositol 145- trisphosphate. KITLG/SCF acts synergistically with other cytokines probably interleukins; Deafness associated genes |  | Mast cells, cell survival and proliferation |
| S100A12 | ENRAGE | P80511 | 9606.ENSP00000357726 | Extracellular newly identified RAGE-binding protein | Extracellular newly identified RAGE-binding protein; S100A12 is a calcium- zinc- and copper-binding protein which plays a prominent role in the regulation of inflammatory processes and immune response. Its proinflammatory activity involves recruitment of leukocytes promotion of cytokine and chemokine production and regulation of leukocyte adhesion and migration. Acts as an alarmin or a danger associated molecular pattern (DAMP) molecule and stimulates innate immune cells via binding to receptor for advanced glycation endproducts (AGER). Binding to AGER activates the MAP-kinase and NF-kappa-B signaling pathways leading to production of proinflammatory cytokines and up-regulation of cell adhesion molecules ICAM1 and VCAM1. Acts as a monocyte and mast cell chemoattractant. Can stimulate mast cell degranulation and activation which generates chemokines histamine and cytokines inducing further leukocyte recruitment to the sites of inflammation. Can inhibit the activity of matrix metalloproteinases; MMP2 MMP3 and MMP9 by chelating Zn(2+) from their active sites. Possesses filariacidal and filariastatic activity. Calcitermin possesses antifungal activity against C.albicans and is also active against E.coli and P.aeruginosa but not L.monocytogenes and S.aureus; EF-hand domain containing |  | leukocyte recruitment, cytokine/chemokine production, monocyte and mast cell attractant and degranulation of mast cells |
| SORT1 | NT3 | Q99523 | 9606.ENSP00000256637 | Neurotensin receptor 3 | Neurotensin receptor 3; Functions as a sorting receptor in the Golgi compartment and as a clearance receptor on the cell surface. Required for protein transport from the Golgi apparatus to the lysosomes by a pathway that is independent of the mannose-6-phosphate receptor (M6PR). Also required for protein transport from the Golgi apparatus to the endosomes. Promotes neuronal apoptosis by mediating endocytosis of the proapoptotic precursor forms of BDNF (proBDNF) and NGFB (proNGFB). Also acts as a receptor for neurotensin. May promote mineralization of the extracellular matrix during osteogenic differentiation by scavenging extracellular LPL. Probably required in adipocytes for the formation of specialized storage vesicles containing the glucose transporter SLC2A4/GLUT4 (GLUT4 storage vesicles or GSVs). These vesicles provide a stable pool of SLC2A4 and confer increased responsiveness to insulin. May also mediate transport from the endoplasmic reticulum to the Golgi; Belongs to the VPS10-related sortilin family. SORT1 subfamily. |  | neuronal apoptosis, protein transport from Golgi to lysosomes |
| CCL28 | CCL28 | Q9NRJ3 | 9606.ENSP00000354416 | Mucosae-associated epithelial chemokine | Mucosae-associated epithelial chemokine; Chemotactic activity for resting CD4 CD8 T-cells and eosinophils. Binds to CCR3 and CCR10 and induces calcium mobilization in a dose-dependent manner; Chemokine ligands | 2 | chemotaxis T helper subsets |
| CST5 | CST5 | P28325 | 9606.ENSP00000307132 | Cystatin D | Cystatin D; Cysteine proteinase inhibitor that possibly plays a protective role against proteinases present in the oral cavity. The order of preference for inhibition is cathepsin S > cathepsin H > cathepsin L > cathepsin B; Cystatins type 2 |  | inhibitor of cysteine proteases, regulation of pro-inflammatory cytokines |
| CX3CL1 | CX3CL1 | P78423 | 9606.ENSP00000006053 | Chemokine (C-X3-C motif) ligand 1 | Chemokine (C-X3-C motif) ligand 1; Acts as a ligand for both CX3CR1 and integrins. Binds to CX3CR1. Binds to integrins ITGAV:ITGB3 and ITGA4:ITGB1. Can activate integrins in both a CX3CR1-dependent and CX3CR1-independent manner. In the presence of CX3CR1 activates integrins by binding to the classical ligand-binding site (site 1) in integrins. In the absence of CX3CR1 binds to a second site (site 2) in integrins which is distinct from site 1 and enhances the binding of other integrin ligands to site 1. The soluble form is chemotactic for T-cells and monocytes and not for neutrophils. The membrane-bound form promotes adhesion of those leukocytes to endothelial cells. May play a role in regulating leukocyte adhesion and migration processes at the endothelium. |  | chemotaxis T helper+monocytes |
| DNER | DNER | Q8NFT8 | 9606.ENSP00000345229 | Delta and Notch-like epidermal growth factor-related receptor | Delta and Notch-like epidermal growth factor-related receptor; Activator of the NOTCH1 pathway. May mediate neuron-glia interaction during astrocytogenesis (By similarity). |  | neuron-glia interaction, growth factor |
| FGF19 | FGF19 | O95750 | 9606.ENSP00000294312 | Fibroblast growth factor 19 | Fibroblast growth factor 19; Involved in the suppression of bile acid biosynthesis through down-regulation of CYP7A1 expression following positive regulation of the JNK and ERK1/2 cascades. Stimulates glucose uptake in adipocytes. Activity requires the presence of KLB and FGFR4; Belongs to the heparin-binding growth factors family. |  | growth factor |
| FLT3LG | Flt3L | P49771 | 9606.ENSP00000469613 | Fms-related tyrosine kinase 3 ligand | Fms-related tyrosine kinase 3 ligand; Stimulates the proliferation of early hematopoietic cells by activating FLT3. Synergizes well with a number of other colony stimulating factors and interleukins; Endogenous ligands |  | Dendritic Cell Development, promotes priming of T cells |
| LIFR | LIFR | P42702 | 9606.ENSP00000263409 | Leukemia inhibitory factor receptor alpha | Leukemia inhibitory factor receptor alpha; Signal-transducing molecule. May have a common pathway with IL6ST. The soluble form inhibits the biological activity of LIF by blocking its binding to receptors on target cells; Belongs to the type I cytokine receptor family. Type 2 subfamily. |  | cytokine receptor family |
| LTA | TNFB | P01374 | 9606.ENSP00000403495 | Tumor necrosis factor ligand superfamily member 1 | Tumor necrosis factor ligand superfamily member 1; Cytokine that in its homotrimeric form binds to TNFRSF1A/TNFR1 TNFRSF1B/TNFBR and TNFRSF14/HVEM. In its heterotrimeric form with LTB binds to TNFRSF3/LTBR. Lymphotoxin is produced by lymphocytes and cytotoxic for a wide range of tumor cells in vitro and in vivo; Tumor necrosis factor superfamily |  | Tumor necros factor ligand superfamily lymphotoxin production, cytotoxic |
| MMP10 | MMP10 | P09238 | 9606.ENSP00000279441 | Matrix metallopeptidase 10 (stromelysin 2) | Matrix metallopeptidase 10 (stromelysin 2); Can degrade fibronectin gelatins of type I III IV and V; weakly collagens III IV and V. Activates procollagenase; Belongs to the peptidase M10A family. |  | Extracellular Matrix Remodeling, process and activate pro-inflammatory cytokines and chemokines |
| PLAU | uPA | P00749 | 9606.ENSP00000361850 | Urokinase-type plasminogen activator | Urokinase-type plasminogen activator; Specifically cleaves the zymogen plasminogen to form the active enzyme plasmin. |  | neutrophils, monocytes, and macrophages, enables infiltration of inflamed tissues, activation of pro-inflammatory cytokines |
| TNFRSF11B | OPG | O00300 | 9606.ENSP00000297350 | Tumor necrosis factor receptor superfamily, member 11b | Tumor necrosis factor receptor superfamily member 11b; Acts as decoy receptor for TNFSF11/RANKL and thereby neutralizes its function in osteoclastogenesis. Inhibits the activation of osteoclasts and promotes osteoclast apoptosis in vitro. Bone homeostasis seems to depend on the local ratio between TNFSF11 and TNFRSF11B. May also play a role in preventing arterial calcification. May act as decoy receptor for TNFSF10/TRAIL and protect against apoptosis. TNFSF10/TRAIL binding blocks the inhibition of osteoclastogenesis; Tumor necrosis factor receptor superfamily |  | tumor necrosis factor receptor family. inhibition of osteoclastogenesis |
| TNFSF12 | TWEAK | O43508 | 9606.ENSP00000293825 | Tumor necrosis factor (ligand) superfamily, member 12 | Tumor necrosis factor (ligand) superfamily member 12; Binds to FN14 and possibly also to TNRFSF12/APO3. Weak inducer of apoptosis in some cell types. Mediates NF-kappa-B activation. Promotes angiogenesis and the proliferation of endothelial cells. Also involved in induction of inflammatory cytokines. Promotes IL8 secretion; Tumor necrosis factor superfamily |  | tumor necrosis factor ligand superfamily, NF-Kappa-B activation |
| CCL1 | CCL1 | P22362 | 9606.ENSP00000225842 | T lymphocyte-secreted protein I-309 | T lymphocyte-secreted protein I-309; Cytokine that is chemotactic for monocytes but not for neutrophils. Binds to CCR8; Belongs to the intercrine beta (chemokine CC) family. | 3 | monocyte chemotaxis |
| FGF21 | FGF21 | Q9NSA1 | 9606.ENSP00000471477 | Fibroblast growth factor 21 | Fibroblast growth factor 21; Stimulates glucose uptake in differentiated adipocytes via the induction of glucose transporter SLC2A1/GLUT1 expression (but not SLC2A4/GLUT4 expression). Activity requires the presence of KLB; Belongs to the heparin-binding growth factors family. |  | metabolism |
| FGF23 | FGF23 | Q9GZV9 | 9606.ENSP00000237837 | Tumor-derived hypophosphatemia-inducing factor | Tumor-derived hypophosphatemia-inducing factor; Regulator of phosphate homeostasis. Inhibits renal tubular phosphate transport by reducing SLC34A1 levels. Upregulates EGR1 expression in the presence of KL (By similarity). Acts directly on the parathyroid to decrease PTH secretion (By similarity). Regulator of vitamin-D metabolism. Negatively regulates osteoblast differentiation and matrix mineralization. |  | metabolism |
| HGF | HGF | Q9UDU6 | 9606.ENSP00000222390 | Hepatocyte growth factor (hepapoietin A | Hepatocyte growth factor (hepapoietin A; scatter factor); Potent mitogen for mature parenchymal hepatocyte cells seems to be a hepatotrophic factor and acts as a growth factor for a broad spectrum of tissues and cell types. Activating ligand for the receptor tyrosine kinase MET by binding to it and promoting its dimerization; Deafness associated genes |  | growth factor |
| IL18R1 | IL18R1 | Q13478 | 9606.ENSP00000387211 | CD218 antigen-like family member A | CD218 antigen-like family member A; Within the IL18 receptor complex responsible for the binding of the proinflammatory cytokine IL18 but not IL1A nor IL1B (Probable). Contributes to IL18-induced cytokine production either independently of SLC12A3 or as a complex with SLC12A3 (By similarity); CD molecules |  | cytokine recptor, proinflammatory |
| IL6 | IL6 | P05231 | 9606.ENSP00000385675 | B-cell stimulatory factor 2 | B-cell stimulatory factor 2; Cytokine with a wide variety of biological functions. It is a potent inducer of the acute phase response. Plays an essential role in the final differentiation of B-cells into Ig- secreting cells Involved in lymphocyte and monocyte differentiation. Acts on B-cells T-cells hepatocytes hematopoietic progenitor cells and cells of the CNS. Required for the generation of T(H)17 cells. Also acts as a myokine. It is discharged into the bloodstream after muscle contraction and acts to increase the breakdown of fats and to improve insulin resistance. It induces myeloma and plasmacytoma growth and induces nerve cells differentiation; Interferons |  | cytokine, proinflammatory |
| OSM | OSM | P13725 | 9606.ENSP00000215781 | Oncostatin M | Oncostatin M; Growth regulator. Inhibits the proliferation of a number of tumor cell lines. Stimulates proliferation of AIDS-KS cells. It regulates cytokine production including IL-6 G-CSF and GM-CSF from endothelial cells. Uses both type I OSM receptor (heterodimers composed of LIPR and IL6ST) and type II OSM receptor (heterodimers composed of OSMR and IL6ST). Involved in the maturation of fetal hepatocytes thereby promoting liver development and regeneration (By similarity). |  | growth regulatory, involved in IL6 regulation |
| TGFA | TGFa | P01135 | 9606.ENSP00000295400 | Protransforming growth factor alpha | Protransforming growth factor alpha; TGF alpha is a mitogenic polypeptide that is able to bind to the EGF receptor/EGFR and to act synergistically with TGF beta to promote anchorage-independent cell proliferation in soft agar. |  | growth factor |
| CCL11 | CCL11 | P51671 | 9606.ENSP00000302234 | Chemokine (C-C motif) ligand 11 | Chemokine (C-C motif) ligand 11; In response to the presence of allergens this protein directly promotes the accumulation of eosinophils a prominent feature of allergic inflammatory reactions. Binds to CCR3; Chemokine ligands | 4 | allergy, eosinophil accumulation |
| CCL2 | MCP1 | P13500 | 9606.ENSP00000225831 | Monocyte chemotactic and activating factor | Monocyte chemotactic and activating factor; Chemotactic factor that attracts monocytes and basophils but not neutrophils or eosinophils. Augments monocyte anti-tumor activity. Has been implicated in the pathogenesis of diseases characterized by monocytic infiltrates like psoriasis rheumatoid arthritis or atherosclerosis. May be involved in the recruitment of monocytes into the arterial wall during the disease process of atherosclerosis; Belongs to the intercrine beta (chemokine CC) family. |  | monocyte chemoattractant |
| CCL25 | CCL25 | O15444 | 9606.ENSP00000375086 | Chemokine (C-C motif) ligand 25 | Chemokine (C-C motif) ligand 25; Potentially involved in T-cell development. Recombinant protein shows chemotactic activity on thymocytes macrophages THP-1 cells and dendritics cells but is inactive on peripheral blood lymphocytes and neutrophils. Binds to CCR9. Isoform 2 is an antagonist of isoform 1. Binds to atypical chemokine receptor ACKR4 and mediates the recruitment of beta-arrestin (ARRB1/2) to ACKR4; Belongs to the intercrine beta (chemokine CC) family. |  | chemokine, inactive for blood lymphocytes |
| CD5 | CD5 | P06127 | 9606.ENSP00000342681 | T-cell surface glycoprotein CD5 | T-cell surface glycoprotein CD5; May act as a receptor in regulating T-cell proliferation; CD molecules |  | regulating T cell proliferation |
| CDCP1 | CDCP1 | Q9H5V8 | 9606.ENSP00000296129 | Subtractive immunization M plus HEp3-associated 135 kDa protein | Subtractive immunization M plus HEp3-associated 135 kDa protein; May be involved in cell adhesion and cell matrix association. May play a role in the regulation of anchorage versus migration or proliferation versus differentiation via its phosphorylation. May be a novel marker for leukemia diagnosis and for immature hematopoietic stem cell subsets. Belongs to the tetraspanin web involved in tumor progression and metastasis; CD molecules |  | cell adhesion |
| CSF1 | CSF1 | P09603 | 9606.ENSP00000327513 | Colony stimulating factor 1 (macrophage) | Colony stimulating factor 1 (macrophage); Cytokine that plays an essential role in the regulation of survival proliferation and differentiation of hematopoietic precursor cells especially mononuclear phagocytes such as macrophages and monocytes. Promotes the release of proinflammatory chemokines and thereby plays an important role in innate immunity and in inflammatory processes. Plays an important role in the regulation of osteoclast proliferation and differentiation the regulation of bone resorption and is required for normal bone development. Required for normal male and female fertility. Promotes reorganization of the actin cytoskeleton regulates formation of membrane ruffles cell adhesion and cell migration. Plays a role in lipoprotein clearance. |  | macrophage and monicyte proliferation and differentiation |
| IL10RB | IL10RB | Q08334 | 9606.ENSP00000290200 | Interleukin-10 receptor subunit beta | Interleukin-10 receptor subunit beta; Shared cell surface receptor required for the activation of five class 2 cytokines: IL10 IL22 IL26 IL28 and IFNL1. The IFNLR1/IL10RB dimer is a receptor for the cytokine ligands IFNL2 and IFNL3 and mediates their antiviral activity. The ligand/receptor complex stimulate the activation of the JAK/STAT signaling pathway leading to the expression of IFN-stimulated genes (ISG) which contribute to the antiviral state. |  | cytokine receptor, JAK-STAT pathway, IFN y stimulation |
| IL15RA | IL15RA | A0A0A0MS77 | 9606.ENSP00000380421 | Interleukin 15 receptor subunit alpha | Interleukin 15 receptor subunit alpha; CD molecules |  | cytokine receptor |
| IL18 | IL18 | Q14116 | 9606.ENSP00000280357 | Interferon gamma-inducing factor | Interferon gamma-inducing factor; Augments natural killer cell activity in spleen cells and stimulates interferon gamma production in T-helper type I cells; Belongs to the IL-1 family. |  | IFN y, cytokine |
| SLAMF1 | SLAMF1 | Q13291 | 9606.ENSP00000306190 | Signaling lymphocytic activation molecule family member 1 | Signaling lymphocytic activation molecule family member 1; Self-ligand receptor of the signaling lymphocytic activation molecule (SLAM) family. SLAM receptors triggered by homo- or heterotypic cell-cell interactions are modulating the activation and differentiation of a wide variety of immune cells and thus are involved in the regulation and interconnection of both innate and adaptive immune response. Activities are controlled by presence or absence of small cytoplasmic adapter proteins SH2D1A/SAP and/or SH2D1B/EAT-2. SLAMF1-induced signal- transduction events in T-lymphocytes are different from those in B-cells. Two modes of SLAMF1 signaling seem to exist: one depending on SH2D1A (and perhaps SH2D1B) and another in which protein-tyrosine phosphatase 2C (PTPN11)-dependent signal transduction operates. Initially it has been proposed that association with SH2D1A prevents binding to inhibitory effectors including INPP5D/SHIP1 and PTPN11/SHP-2. However signaling is also regulated by SH2D1A which can simultaneously interact with and recruit FYN which subsequently phosphorylates and activates SLAMF1. Mediates IL-2-independent proliferation of activated T-cells during immune responses and induces IFN-gamma production (By similarity). Downstreaming signaling involves INPP5D DOK1 and DOK2 leading to inhibited IFN-gamma production in T-cells and PRKCQ BCL10 and NFKB1 leading to increased T-cell activation and Th2 cytokine production (By similarity). Promotes T-cell receptor-induced IL-4 secretion by CD4(+) cells (By similarity). Inhibits antigen receptor-mediated production of IFN-gamma but not IL-2 in CD4(-)/CD8(-) T-cells (By similarity). Required for IL-4 production by germinal centers T follicular helper (T(Fh))cells (By similarity). May inhibit CD40-induced signal transduction in monocyte-derived dendritic cells. May play a role in a allergic responses and may regulate allergen-induced Th2 cytokine and Th1 cytokine secretion (By similarity). In conjunction with SLAMF6 controls the transition between positive selection and the subsequent expansion and differentiation of the thymocytic natural killer T (NKT) cell lineage. Involved in the peripheral differentiation of indifferent natural killer T (iNKT) cells toward a regulatory NKT2 type (By similarity). In macrophages involved in down-regulation of IL-12 TNF-alpha and nitric oxide in response to lipopolysaccharide (LPS) (By similarity). In B-cells activates the ERK signaling pathway independently of SH2D1A but implicating both SYK and INPP5D and activates Akt signaling dependent on SYK and SH2D1A (By similarity). In B-cells also activates p38 MAPK and JNK1 and JNK2. In conjunction with CD84/SLAMF5 and SLAMF6 may be a negative regulator of the humoral immune response (By similarity). |  | immune cell activation/differentiation, AKT signalling, humoral immunity regulation |
| TNF | TNF | P01375 | 9606.ENSP00000398698 | Tumor necrosis factor ligand superfamily member 2 | Tumor necrosis factor ligand superfamily member 2; Cytokine that binds to TNFRSF1A/TNFR1 and TNFRSF1B/TNFBR. It is mainly secreted by macrophages and can induce cell death of certain tumor cell lines. It is potent pyrogen causing fever by direct action or by stimulation of interleukin-1 secretion and is implicated in the induction of cachexia Under certain conditions it can stimulate cell proliferation and induce cell differentiation. Impairs regulatory T-cells (Treg) function in individuals with rheumatoid arthritis via FOXP3 dephosphorylation. Upregulates the expression of protein phosphatase 1 (PP1) which dephosphorylates the key 'Ser-418' residue of FOXP3 thereby inactivating FOXP3 and rendering Treg cells functionally defective. Key mediator of cell death in the anticancer action of BCG-stimulated neutrophils in combination with DIABLO/SMAC mimetic in the RT4v6 bladder cancer cell line; Tumor necrosis factor superfamily |  | TNF superfamily; anti tumoral effects, impairs Tregulatory cells |
| TNFSF10 | CD253 | P50591 | 9606.ENSP00000241261 | Tumor necrosis factor (ligand) superfamily, member 10 | Tumor necrosis factor (ligand) superfamily member 10; Cytokine that binds to TNFRSF10A/TRAILR1 TNFRSF10B/TRAILR2 TNFRSF10C/TRAILR3 TNFRSF10D/TRAILR4 and possibly also to TNFRSF11B/OPG. Induces apoptosis. Its activity may be modulated by binding to the decoy receptors TNFRSF10C/TRAILR3 TNFRSF10D/TRAILR4 and TNFRSF11B/OPG that cannot induce apoptosis; CD molecules |  | induce apoptosis, activation of NK cells, macrophages, and T cells |
| VEGFA | VEGFA | Q9H1W9 | 9606.ENSP00000478570 | Vascular endothelial growth factor A | Vascular endothelial growth factor A; Growth factor active in angiogenesis vasculogenesis and endothelial cell growth. Induces endothelial cell proliferation promotes cell migration inhibits apoptosis and induces permeabilization of blood vessels. Binds to the FLT1/VEGFR1 and KDR/VEGFR2 receptors heparan sulfate and heparin. NRP1/Neuropilin-1 binds isoforms VEGF-165 and VEGF-145. Isoform VEGF165B binds to KDR but does not activate downstream signaling pathways does not activate angiogenesis and inhibits tumor growth. Binding to NRP1 receptor initiates a signaling pathway needed for motor neuron axon guidance and cell body migration including for the caudal migration of facial motor neurons from rhombomere 4 to rhombomere 6 during embryonic development (By similarity). |  | growth factor (endothelial cells), apoptosis inhibition, |
| CD6 | CD6 | P30203 | 9606.ENSP00000323280 | T-cell differentiation antigen CD6 | T-cell differentiation antigen CD6; Cell adhesion molecule that mediates cell-cell contacts and regulates T-cell responses via its interaction with ALCAM/CD166. Contributes to signaling cascades triggered by activation of the TCR/CD3 complex. Functions as costimulatory molecule; promotes T-cell activation and proliferation. Contributes to the formation and maturation of the immunological synapse. Functions as calcium-dependent pattern receptor that binds and aggregates both Gram-positive and Gram-negative bacteria. Binds both lipopolysaccharide (LPS) from Gram-negative bacteria and lipoteichoic acid from Gram-positive bacteria. LPS binding leads to the activation of signaling cascades and down-stream MAP kinases. Mediates activation of the inflammatory response and the secretion of pro-inflammatory cytokines in response to LPS. | 5 | T-cell response regulation and proliferation, inflammation mediator in response to LPS |
| CXCL10 | CXCL10 | P02778 | 9606.ENSP00000305651 | 10 kDa interferon gamma-induced protein | 10 kDa interferon gamma-induced protein; Chemotactic for monocytes and T-lymphocytes. Binds to CXCR3; Belongs to the intercrine alpha (chemokine CxC) family. |  | chemotaxis (monocytes and T cells) |
| CXCL9 | CXCL9 | Q07325 | 9606.ENSP00000354901 | Monokine induced by interferon-gamma | Monokine induced by interferon-gamma; Cytokine that affects the growth movement or activation state of cells that participate in immune and inflammatory response. Chemotactic for activated T-cells. Binds to CXCR3; Belongs to the intercrine alpha (chemokine CxC) family. |  | chemotas of T cells, monokine |
| IFNG | IFNg | P01579 | 9606.ENSP00000229135 | Immune interferon | Immune interferon; Produced by lymphocytes activated by specific antigens or mitogens. IFN-gamma in addition to having antiviral activity has important immunoregulatory functions. It is a potent activator of macrophages it has antiproliferative effects on transformed cells and it can potentiate the antiviral and antitumor effects of the type I interferons; Belongs to the type II (or gamma) interferon family. |  | interferon, cytokine activation of macrophages |
| IL12B | IL12B | P29460 | 9606.ENSP00000231228 | Cytotoxic lymphocyte maturation factor 40 kDa subunit | Cytotoxic lymphocyte maturation factor 40 kDa subunit; Cytokine that can act as a growth factor for activated T and NK cells enhance the lytic activity of NK/lymphokine- activated killer cells and stimulate the production of IFN-gamma by resting PBMC; Belongs to the type I cytokine receptor family. Type 3 subfamily. |  | growth factor (T cells and NK cells) |
| TNFRSF9 | TNFRSF9 | Q07011 | 9606.ENSP00000478699 | Tumor necrosis factor receptor superfamily, member 9 | Tumor necrosis factor receptor superfamily member 9; Receptor for TNFSF9/4-1BBL. Possibly active during T cell activation; CD molecules |  | TNF family; T cell activation |
| TNFSF11 | CD254 | O14788 | 9606.ENSP00000381775 | Tumor necrosis factor (ligand) superfamily, member 11 | Tumor necrosis factor (ligand) superfamily member 11; Cytokine that binds to TNFRSF11B/OPG and to TNFRSF11A/RANK. Osteoclast differentiation and activation factor. Augments the ability of dendritic cells to stimulate naive T-cell proliferation. May be an important regulator of interactions between T-cells and dendritic cells and may play a role in the regulation of the T-cell-dependent immune response. May also play an important role in enhanced bone-resorption in humoral hypercalcemia of malignancy. Induces osteoclastogenesis by activating multiple signaling pathways in osteoclast precursor cells chief among which is induction of long lasting oscillations in the intracellular concentration of Ca (2+) resulting in the activation of NFATC1 which translocates to the nucleus and induces osteoclast-specific gene transcription to allow differentiation of osteoclasts. During osteoclast differentiation in a TMEM64 and ATP2A2-dependent manner induces activation of CREB1 and mitochondrial ROS generation necessary for proper osteoclast generation (By similarity); Belongs to the tumor necrosis factor family. |  | TNF family; T cell activation |
| ADA | ADA | P00813 | 9606.ENSP00000361965 | Adenosine aminohydrolase | Adenosine aminohydrolase; Catalyzes the hydrolytic deamination of adenosine and 2- deoxyadenosine. Plays an important role in purine metabolism and in adenosine homeostasis. Modulates signaling by extracellular adenosine and so contributes indirectly to cellular signaling events. Acts as a positive regulator of T-cell coactivation by binding DPP4. Its interaction with DPP4 regulates lymphocyte- epithelial cell adhesion. Enhances dendritic cell immunogenicity by affecting dendritic cell costimulatory molecule expression and cytokines and chemokines secretion (By similarity). Enhances CD4+ T-cell differentiation and proliferation. Acts as a positive modulator of adenosine receptors ADORA1 and ADORA2A by enhancing their ligand affinity via conformational change. Stimulates plasminogen activation. Plays a role in male fertility. Plays a protective role in early postimplantation embryonic development (By similarity); Adenosine deaminase family | 6 | purine metabolism, T -cell coactivation, dendritic cell stimulation, |
| AXIN1 | AXIN1 | O15169 | 9606.ENSP00000262320 | Axis inhibition protein 1 | Axis inhibition protein 1; Component of the beta-catenin destruction complex required for regulating CTNNB1 levels through phosphorylation and ubiquitination and modulating Wnt-signaling. Controls dorsoventral patterning via two opposing effects; down-regulates CTNNB1 to inhibit the Wnt signaling pathway and ventralize embryos but also dorsalizes embryos by activating a Wnt-independent JNK signaling pathway. In Wnt signaling probably facilitates the phosphorylation of CTNNB1 and APC by GSK3B. Likely to function as a tumor suppressor. Enhances TGF-beta signaling by recruiting the RNF111 E3 ubiquitin ligase and promoting the degradation of inhibitory SMAD7. Also component of the AXIN1-HIPK2-TP53 complex which controls cell growth apoptosis and development. Facilitates the phosphorylation of TP53 by HIPK2 upon ultraviolet irradiation; Protein phosphatase 1 regulatory subunits |  | WNT signalling, tumor supression, |
| CASP8 | CASP8 | Q9UQ81 | 9606.ENSP00000351273 | Caspase 8, apoptosis-related cysteine peptidase | Caspase 8 apoptosis-related cysteine peptidase; Most upstream protease of the activation cascade of caspases responsible for the TNFRSF6/FAS mediated and TNFRSF1A induced cell death. Binding to the adapter molecule FADD recruits it to either receptor. The resulting aggregate called death- inducing signaling complex (DISC) performs CASP8 proteolytic activation. The active dimeric enzyme is then liberated from the DISC and free to activate downstream apoptotic proteases. Proteolytic fragments of the N-terminal propeptide (termed CAP3 CAP5 and CAP6) are likely retained in the DISC. Cleaves and activates CASP3 CASP4 CASP6 CASP7 CASP9 and CASP10. May participate in the GZMB apoptotic pathways. Cleaves ADPRT. Hydrolyzes the small-molecule substrate Ac-Asp-Glu-Val-Asp-\|-AMC. Likely target for the cowpox virus CRMA death inhibitory protein. Isoform 5 isoform 6 isoform 7 and isoform 8 lack the catalytic site and may interfere with the pro-apoptotic activity of the complex. |  | cell death, proteolytic activation, apoptosis |
| CCL13 | MCP4 | Q99616 | 9606.ENSP00000225844 | Monocyte chemoattractant protein 4 | Monocyte chemoattractant protein 4; Chemotactic factor that attracts monocytes lymphocytes basophils and eosinophils but not neutrophils. Signals through CCR2B and CCR3 receptors. Plays a role in the accumulation of leukocytes at both sides of allergic and non-allergic inflammation. May be involved in the recruitment of monocytes into the arterial wall during the disease process of atherosclerosis. May play a role in the monocyte attraction in tissues chronically exposed to exogenous pathogens; Chemokine ligands |  | leukocyte activation, chemoattraction (particularly monocytes) |
| CCL3 | CCL3 | P10147 | 9606.ENSP00000477908 | Macrophage inflammatory protein 1-alpha | Macrophage inflammatory protein 1-alpha; Monokine with inflammatory and chemokinetic properties. Binds to CCR1 CCR4 and CCR5. One of the major HIV-suppressive factors produced by CD8+ T-cells. Recombinant MIP-1-alpha induces a dose-dependent inhibition of different strains of HIV-1 HIV-2 and simian immunodeficiency virus (SIV); Belongs to the intercrine beta (chemokine CC) family. |  | inflammation, chemotaxis (particularly monocytes) |
| CCL4 | CCL4 | P13236 | 9606.ENSP00000482259 | Macrophage inflammatory protein 1-beta | Macrophage inflammatory protein 1-beta; Monokine with inflammatory and chemokinetic properties. Binds to CCR5. One of the major HIV-suppressive factors produced by CD8+ T-cells. Recombinant MIP-1-beta induces a dose-dependent inhibition of different strains of HIV-1 HIV-2 and simian immunodeficiency virus (SIV). The processed form MIP-1-beta(3-69) retains the abilities to induce down-modulation of surface expression of the chemokine receptor CCR5 and to inhibit the CCR5- mediated entry of HIV-1 in T-cells. MIP-1-beta(3-69) is also a ligand for CCR1 and CCR2 isoform B; Belongs to the intercrine beta (chemokine CC) family. |  | inflammation, chemotaxis (particularly monocytes) |
| CCL8 | MCP2 | P80075 | 9606.ENSP00000378118 | Monocyte chemoattractant protein 2 | Monocyte chemoattractant protein 2; Chemotactic factor that attracts monocytes lymphocytes basophils and eosinophils. May play a role in neoplasia and inflammatory host responses. This protein can bind heparin. The processed form MCP-2(6-76) does not show monocyte chemotactic activity but inhibits the chemotactic effect most predominantly of CCL7 and also of CCL2 and CCL5 and CCL8; Belongs to the intercrine beta (chemokine CC) family. |  | inhibition of chemotaxis (particularly monocytes) |
| CD244 | CD244 | Q9BZW8 | 9606.ENSP00000357012 | CD244 molecule, natural killer cell receptor 2B4 | CD244 molecule natural killer cell receptor 2B4; Heterophilic receptor of the signaling lymphocytic activation molecule (SLAM) family; its ligand is CD48. SLAM receptors triggered by homo- or heterotypic cell-cell interactions are modulating the activation and differentiation of a wide variety of immune cells and thus are involved in the regulation and interconnection of both innate and adaptive immune response. Activities are controlled by presence or absence of small cytoplasmic adapter proteins SH2D1A/SAP and/or SH2D1B/EAT-2. Acts as activating natural killer (NK) cell receptor. Activating function implicates association with SH2D1A and FYN. Downstreaming signaling involves predominantly VAV1 and to a lesser degree INPP5D/SHIP1 and CBL. Signal attenuation in the absence of SH2D1A is proposed to be dependent on INPP5D and to a lesser extent PTPN6/SHP-1 and PTPN11/SHP-2. Stimulates NK cell cytotoxicity production of IFN-gamma and granule exocytosis. Optimal expansion and activation of NK cells seems to be dependent on the engagement of CD244 with CD48 expressed on neighboring NK cells (By similarity). Acts as costimulator in NK activation by enhancing signals by other NK receptors such as NCR3 and NCR1. At early stages of NK cell differentiation may function as an inhibitory receptor possibly ensuring the self- tolerance of developing NK cells. Involved in the regulation of CD8(+) T-cell proliferation; expression on activated T-cells and binding to CD488 provides costimulatory-like function for neighboring T-cells (By similarity). Inhibits inflammatory responses in dendritic cells (DCs) (By similarity). |  | NK cell receptor(activation) |
| CD40 | CD40 | P25942 | 9606.ENSP00000361359 | Tumor necrosis factor receptor superfamily member 5 | Tumor necrosis factor receptor superfamily member 5; Receptor for TNFSF5/CD40LG. Transduces TRAF6- and MAP3K8-mediated signals that activate ERK in macrophages and B cells leading to induction of immunoglobulin secretion. |  | TNF superfamily, ERK activation,immunoglobulin secretion |
| CXCL1 | CXCL1 | P09341 | 9606.ENSP00000379110 | Chemokine (C-X-C motif) ligand 1 (melanoma growth stimulating activity, alpha) | Chemokine (C-X-C motif) ligand 1 (melanoma growth stimulating activity alpha); Has chemotactic activity for neutrophils. May play a role in inflammation and exerts its effects on endothelial cells in an autocrine fashion. In vitro the processed forms GRO- alpha(4-73) GRO-alpha(5-73) and GRO-alpha(6-73) show a 30-fold higher chemotactic activity; Chemokine ligands |  | neutrophil chemotaxis, inflammation, |
| CXCL11 | CXCL11 | O14625 | 9606.ENSP00000306884 | Interferon-inducible T-cell alpha chemoattractant | Interferon-inducible T-cell alpha chemoattractant; Chemotactic for interleukin-activated T-cells but not unstimulated T-cells neutrophils or monocytes. Induces calcium release in activated T-cells. Binds to CXCR3. May play an important role in CNS diseases which involve T-cell recruitment. May play a role in skin immune responses; Belongs to the intercrine alpha (chemokine CxC) family. |  | chemotaxis(activated T cells), CNS recruitment of T cells |
| CXCL5 | CXCL5 | P42830 | 9606.ENSP00000296027 | Epithelial-derived neutrophil-activating protein 78 | Epithelial-derived neutrophil-activating protein 78; Involved in neutrophil activation. In vitro ENA-78(8- 78) and ENA-78(9-78) show a threefold higher chemotactic activity for neutrophil granulocytes; Chemokine ligands |  | neutrophil activation and chemotaxis |
| CXCL6 | CXCL6 | P80162 | 9606.ENSP00000226317 | Granulocyte chemotactic protein 2 | Granulocyte chemotactic protein 2; Chemotactic for neutrophil granulocytes. Signals through binding and activation of its receptors (CXCR1 and CXCR2). In addition to its chemotactic and angiogenic properties it has strong antibacterial activity against Gram-positive and Gram- negative bacteria (90-fold-higher when compared to CXCL5 and CXCL7); Chemokine ligands |  | antibacterial, chemotaxis angiogenesis |
| CXCL8 | IL8 | Q9UCS0 | 9606.ENSP00000306512 | Monocyte-derived neutrophil chemotactic factor | Monocyte-derived neutrophil chemotactic factor; IL-8 is a chemotactic factor that attracts neutrophils basophils and T-cells but not monocytes. It is also involved in neutrophil activation. It is released from several cell types in response to an inflammatory stimulus. IL-8(6-77) has a 5-10-fold higher activity on neutrophil activation IL-8(5-77) has increased activity on neutrophil activation and IL-8(7-77) has a higher affinity to receptors CXCR1 and CXCR2 as compared to IL-8(1-77) respectively; Chemokine ligands |  | chemotaxis (neutrophil, basophil and T-cell attraction) |
| EIF4EBP1 | EIF4EBP1 | Q13541 | 9606.ENSP00000340691 | Phosphorylated heat- and acid-stable protein regulated by insulin 1 | Phosphorylated heat- and acid-stable protein regulated by insulin 1; Repressor of translation initiation that regulates EIF4E activity by preventing its assembly into the eIF4F complex: hypophosphorylated form competes with EIF4G1/EIF4G3 and strongly binds to EIF4E leading to repress translation. In contrast hyperphosphorylated form dissociates from EIF4E allowing interaction between EIF4G1/EIF4G3 and EIF4E leading to initiation of translation. Mediates the regulation of protein translation by hormones growth factors and other stimuli that signal through the MAP kinase and mTORC1 pathways. |  | protein translation |
| IL7 | IL7 | P13232 | 9606.ENSP00000263851 | Interleukin 7 | Interleukin 7; Hematopoietic growth factor capable of stimulating the proliferation of lymphoid progenitors. It is important for proliferation during certain stages of B-cell maturation; Belongs to the IL-7/IL-9 family. |  | growth factor, proliferation |
| MMP1 | MMP1 | P03956 | 9606.ENSP00000322788 | Matrix metallopeptidase 1 (interstitial collagenase) | Matrix metallopeptidase 1 (interstitial collagenase); Cleaves collagens of types I II and III at one site in the helical domain. Also cleaves collagens of types VII and X. In case of HIV infection interacts and cleaves the secreted viral Tat protein leading to a decrease in neuronal Tat's mediated neurotoxicity; Endogenous ligands |  | neurotoxicity |
| PDCD1 | PDCD1 | Q15116 | 9606.ENSP00000335062 | Programmed cell death protein 1 | Programmed cell death protein 1; Inhibitory cell surface receptor involved in the regulation of T-cell function during immunity and tolerance. Upon ligand binding inhibits T-cell effector functions in an antigen- specific manner. Possible cell death inducer in association with other factors; CD molecules |  | inhibtis T effector cell function |
| SIRT2 | SIRT2 | Q8IXJ6 | 9606.ENSP00000249396 | NAD-dependent protein deacetylase sirtuin-2 | NAD-dependent protein deacetylase sirtuin-2; NAD-dependent protein deacetylase which deacetylates internal lysines on histone and alpha-tubulin as well as many other proteins such as key transcription factors. Participates in the modulation of multiple and diverse biological processes such as cell cycle control genomic integrity microtubule dynamics cell differentiation metabolic networks and autophagy. Plays a major role in the control of cell cycle progression and genomic stability. Functions in the antephase checkpoint preventing precocious mitotic entry in response to microtubule stress agents and hence allowing proper inheritance of chromosomes. Positively regulates the anaphase promoting complex/cyclosome (APC/C) ubiquitin ligase complex activity by deacetylating CDC20 and FZR1 then allowing progression through mitosis. Associates both with chromatin at transcriptional start sites (TSSs) and enhancers of active genes. Plays a role in cell cycle and chromatin compaction through epigenetic modulation of the regulation of histone H4 'Lys-20' methylation (H4K20me1) during early mitosis. Specifically deacetylates histone H4 at 'Lys-16' (H4K16ac) between the G2/M transition and metaphase enabling H4K20me1 deposition by KMT5A leading to ulterior levels of H4K20me2 and H4K20me3 deposition throughout cell cycle and mitotic S-phase progression. Deacetylates KMT5A modulating KMT5A chromatin localization during the mitotic stress response. Deacetylates also histone H3 at 'Lys-57' (H3K56ac) during the mitotic G2/M transition. Upon bacterium Listeria monocytogenes infection deacetylates 'Lys-18' of histone H3 in a receptor tyrosine kinase MET- and PI3K/Akt-dependent manner thereby inhibiting transcriptional activity and promoting late stages of listeria infection. During oocyte meiosis progression may deacetylate histone H4 at 'Lys-16' (H4K16ac) and alpha-tubulin regulating spindle assembly and chromosome alignment by influencing microtubule dynamics and kinetochore function. Deacetylates histone H4 at 'Lys-16' (H4K16ac) at the VEGFA promoter and thereby contributes to regulate expression of VEGFA a key regulator of angiogenesis. Deacetylates alpha-tubulin at 'Lys-40' and hence controls neuronal motility oligodendroglial cell arbor projection processes and proliferation of non-neuronal cells. Phosphorylation at Ser-368 by a G1/S-specific cyclin E-CDK2 complex inactivates SIRT2-mediated alpha-tubulin deacetylation negatively regulating cell adhesion cell migration and neurite outgrowth during neuronal differentiation. Deacetylates PARD3 and participates in the regulation of Schwann cell peripheral myelination formation during early postnatal development and during postinjury remyelination. Involved in several cellular metabolic pathways. Plays a role in the regulation of blood glucose homeostasis by deacetylating and stabilizing phosphoenolpyruvate carboxykinase PCK1 activity in response to low nutrient availability. Acts as a key regulator in the pentose phosphate pathway (PPP) by deacetylating and activating the glucose-6-phosphate G6PD enzyme and therefore stimulates the production of cytosolic NADPH to counteract oxidative damage. Maintains energy homeostasis in response to nutrient deprivation as well as energy expenditure by inhibiting adipogenesis and promoting lipolysis. Attenuates adipocyte differentiation by deacetylating and promoting FOXO1 interaction to PPARG and subsequent repression of PPARG-dependent transcriptional activity. Plays a role in the regulation of lysosome-mediated degradation of protein aggregates by autophagy in neuronal cells. Deacetylates FOXO1 in response to oxidative stress or serum deprivation thereby negatively regulating FOXO1- mediated autophagy. Deacetylates a broad range of transcription factors and co-regulators regulating target gene expression. Deacetylates transcriptional factor FOXO3 stimulating the ubiquitin ligase SCF(SKP2)-mediated FOXO3 ubiquitination and degradation (By similarity). Deacetylates HIF1A and therefore promotes HIF1A degradation and inhibition of HIF1A transcriptional activity in tumor cells in response to hypoxia. Deacetylates RELA in the cytoplasm inhibiting NF-kappaB-dependent transcription activation upon TNF-alpha stimulation. Inhibits transcriptional activation by deacetylating p53/TP53 and EP300. Deacetylates also EIF5A. Functions as a negative regulator on oxidative stress-tolerance in response to anoxia-reoxygenation conditions. Plays a role as tumor suppressor; Belongs to the sirtuin family. Class I subfamily. |  | metabolism, cell differentiation, autophagy |
| STAMBP | STAMBP | O95630 | 9606.ENSP00000377633 | Associated molecule with the SH3 domain of STAM | Associated molecule with the SH3 domain of STAM; Zinc metalloprotease that specifically cleaves 'Lys-63'- linked polyubiquitin chains. Does not cleave 'Lys-48'-linked polyubiquitin chains (By similarity). Plays a role in signal transduction for cell growth and MYC induction mediated by IL-2 and GM-CSF. Potentiates BMP (bone morphogenetic protein) signaling by antagonizing the inhibitory action of SMAD6 and SMAD7. Has a key role in regulation of cell surface receptor-mediated endocytosis and ubiquitin-dependent sorting of receptors to lysosomes. Endosomal localization of STAMBP is required for efficient EGFR degradation but not for its internalization (By similarity). Involved in the negative regulation of PI3K-AKT-mTOR and RAS-MAP signaling pathways. |  | cell growth, endocytosis |
| SULT1A1 | ST1A1 | P50225 | 9606.ENSP00000378972 | Sulfotransferase family, cytosolic, 1A, phenol-preferring, member 1 | Sulfotransferase family cytosolic 1A phenol-preferring member 1; Sulfotransferase that utilizes 3'-phospho-5'-adenylyl sulfate (PAPS) as sulfonate donor to catalyze the sulfate conjugation of catecholamines phenolic drugs and neurotransmitters. Has also estrogen sulfotransferase activity. responsible for the sulfonation and activation of minoxidil. Is Mediates the metabolic activation of carcinogenic N- hydroxyarylamines to DNA binding products and could so participate as modulating factor of cancer risk; Sulfotransferases cytosolic |  | cancerogenic metabolism |
| TGFB1 | TGFb1 | P01137 | 9606.ENSP00000221930 | Transforming growth factor, beta 1 | Transforming growth factor beta 1; Multifunctional protein that controls proliferation differentiation and other functions in many cell types. Many cells synthesize TGFB1 and have specific receptors for it. It positively and negatively regulates many other growth factors. It plays an important role in bone remodeling as it is a potent stimulator of osteoblastic bone formation causing chemotaxis proliferation and differentiation in committed osteoblasts (By similarity). Stimulates sustained production of collagen through the activation of CREB3L1 by regulated intramembrane proteolysis (RIP). Can promote either T-helper 17 cells (Th17) or regulatory T-cells (Treg) lineage differentiation in a concentration-dependent manner. At high concentrations leads to FOXP3-mediated suppression of RORC and down-regulation of IL-17 expression favoring Treg cell development. At low concentrations in concert with IL-6 and IL-21 leads to expression of the IL-17 and IL-23 receptors favoring differentiation to Th17 cells. Mediates SMAD2/3 activation by inducing its phosphorylation and subsequent translocation to the nucleus. Can induce epithelial-to-mesenchymal transition (EMT) and cell migration in various cell types; Endogenous ligands |  | proliferation differentiation, Treg (high concentration), Th17(low concentrations) |
| TNFSF14 | TNFSF14 | O43557 | 9606.ENSP00000469049 | Tumor necrosis factor (ligand) superfamily, member 14 | Tumor necrosis factor (ligand) superfamily member 14; Cytokine that binds to TNFRSF3/LTBR. Binding to the decoy receptor TNFRSF6B modulates its effects. Activates NFKB stimulates the proliferation of T-cells and inhibits growth of the adenocarcinoma HT-29. Acts as a receptor for Herpes simplex virus; CD molecules |  | TNF superfamily, NFKB stimulation |
| CCL20 | CCL20 | P78556 | 9606.ENSP00000351671 | Liver and activation-regulated chemokine | Liver and activation-regulated chemokine; Acts as a ligand for C-C chemokine receptor CCR6. Signals through binding and activation of CCR6 and induces a strong chemotactic response and mobilization of intracellular calcium ions. The ligand-receptor pair CCL20-CCR6 is responsible for the chemotaxis of dendritic cells (DC) effector/memory T-cells and B- cells and plays an important role at skin and mucosal surfaces under homeostatic and inflammatory conditions as well as in pathology including cancer and various autoimmune diseases. CCL20 acts as a chemotactic factor that attracts lymphocytes and slightly neutrophils but not monocytes. Involved in the recruitment of both the proinflammatory IL17 producing helper T-cells (Th17) and the regulatory T-cells (Treg) to sites of inflammation. Required for optimal migration of thymic natural regulatory T cells (nTregs) and DN1 early thymocyte progenitor cells (By similarity). C-terminal processed forms have been shown to be equally chemotactically active for leukocytes. Positively regulates sperm motility and chemotaxis via its binding to CCR6 which triggers Ca2+ mobilization in the sperm which is important for its motility. Inhibits proliferation of myeloid progenitors in colony formation assays. May be involved in formation and function of the mucosal lymphoid tissues by attracting lymphocytes and dendritic cells towards epithelial cells (By similarity). Possesses antibacterial activity towards E.coli ATCC 25922 and S.aureus ATCC 29213. | 7 | recruitment of Th17 and Tregs to site sof inflammation |
| IL10 | IL10 | P22301 | 9606.ENSP00000412237 | Cytokine synthesis inhibitory factor | Cytokine synthesis inhibitory factor; Inhibits the synthesis of a number of cytokines including IFN-gamma IL-2 IL-3 TNF and GM-CSF produced by activated macrophages and by helper T-cells; Belongs to the IL-10 family. |  | anti-inflammatory |
| IL17A | IL-17A | Q16552 | 9606.ENSP00000344192 | Cytotoxic T-lymphocyte-associated antigen 8 | Cytotoxic T-lymphocyte-associated antigen 8; Ligand for IL17RA and IL17RC. The heterodimer formed by IL17A and IL17F is a ligand for the heterodimeric complex formed by IL17RA and IL17RC. Involved in inducing stromal cells to produce proinflammatory and hematopoietic cytokines; Belongs to the IL-17 family. |  | inflammation |
| IL17C | IL-17C | Q9P0M4 | 9606.ENSP00000244241 | Interleukin 17C | Interleukin 17C; Cytokine that plays a crucial role in innate immunity of the epithelium including to intestinal bacterial pathogens in an autocrine manner. Stimulates the production of antibacterial peptides and proinflammatory molecules for host defense by signaling through the NF-kappa-B and MAPK pathways. Acts synergically with IL22 in inducing the expression of antibacterial peptides including S100A8 S100A9 REG3A and REG3G. Synergy is also observed with TNF and IL1B in inducing DEFB2 from keratinocytes. Depending on the type of insult may have both protective and pathogenic properties either by maintaining epithelial homeostasis after an inflammatory challenge or by promoting inflammatory phenotype. Enhanced IL17C/IL17RE signaling may also lead to greater susceptibility to autoimmune diseases; Belongs to the IL-17 family. |  | inflammation, NF kappa B, MAPK |

| **Supplementary Table 3.** Adjusted models. **A.** Effect of bioytpe when participants with immunomodulatory medication were excluded (right side). **B.** Effect of sex and biotype when sex was included in the model (right side) | | | | | | | |
| --- | --- | --- | --- | --- | --- | --- | --- |
| **A. Sensitivity analysis** | **Cluster** | **Main model: biotype+site** | | | **Adjusted model*: biotype+site** | | |
|  | **Cluster** | **Pillai's Trace Test** | **F** | **p-value** | **Pillai's Trace Test** | **F** | **p-value** |
| HIP vs. LIP | **Cluster 1** | 0.10 | 2.50 | **0.034** | 0.11 | 2.52 | **0.034** |
|  | **Cluster 2** | 0.26 | 3.69 | **< 0.001** | 0.27 | 3.13 | **< 0.001** |
|  | **Cluster 3** | 0.34 | 7.33 | **< 0.001** | 0.37 | 7.47 | **< 0.001** |
|  | **Cluster 4** | 0.33 | 4.02 | **< 0.001** | 0.34 | 3.90 | **< 0.001** |
|  | **Cluster 5** | 0.10 | 1.82 | 0.089 | 0.07 | 1.29 | 0.260 |
|  | **Cluster 6** | 0.87 | 29.64 | **< 0.001** | 0.87 | 26.44 | **< 0.001** |
|  | **Cluster 7** | 0.11 | 3.55 | **0.009** | 0.09 | 2.98 | **0.022** |
| * Main model exlcuding participants who were on any potentially immunomodulatory medication | | | | | | |  |
| **B. Effect of sex** | **Cluster** | **Main model: biotype+site** | | | **Adjusted model*: biotype+site+sex** | | |
|  | **Cluster** | **Pillai's Trace Test** | **F** | **p-value** | **Pillai's Trace Test** | **F** | **p-value** |
| HIP vs. LIP | **Cluster 1** | 0.10 | 2.50 | **0.034** | 0.10 | 2.47 | **0.036** |
|  | **Cluster 2** | 0.26 | 3.69 | **< 0.001** | 0.29 | 3.60 | **< 0.001** |
|  | **Cluster 3** | 0.34 | 7.33 | **< 0.001** | 0.35 | 7.39 | **< 0.001** |
|  | **Cluster 4** | 0.33 | 4.02 | **< 0.001** | 0.34 | 4.10 | **< 0.001** |
|  | **Cluster 5** | 0.10 | 1.82 | 0.089 | 0.11 | 1.85 | 0.085 |
|  | **Cluster 6** | 0.87 | 29.64 | **< 0.001** | 0.88 | 28.70 | **< 0.001** |
|  | **Cluster 7** | 0.11 | 3.55 | **0.009** | 0.11 | 3.43 | **0.011** |
| Effect of sex | **Cluster 1** | na | na | na | 0.04 | 0.89 | 0.488 |
|  | **Cluster 2** | na | na | na | 0.22 | 2.45 | **0.007** |
|  | **Cluster 3** | na | na | na | 0.18 | 2.99 | **0.004** |
|  | **Cluster 4** | na | na | na | 0.18 | 1.75 | 0.061 |
|  | **Cluster 5** | na | na | na | 0.14 | 2.45 | **0.022** |
|  | **Cluster 6** | na | na | na | 0.37 | 2.34 | **0.002** |
|  | **Cluster 7** | na | na | na | 0.03 | 0.75 | 0.562 |
| Abbreviations: na = not applicable | | |  |  |  |  |  |

| **Supplementary Table 4a.** Additional sample characteristics between HIP and LIP groups. | | | | |  |
| --- | --- | --- | --- | --- | --- |
|  |  |  |  |  |  |
| **Demographic** | **Overall** | **HIP** | **LIP** | **p-value** |  |
| **Characteristics** | **N = 126** | **N = 53** | **N = 73** |  |  |
| **Study site**^1b^ |  |  |  | **<0.001**^2a^ |  |
| Barcelona | 43 (34%) | 11 (21%) | 32 (44%) |  |  |
| Budapest | 41 (33%) | 3 (5.7%) | 38(52%) |  |  |
| Frankfurt | 42 (33%) | 39 (74%) | 3 (4.1%) |  |  |
| **Sex**^1b^ |  |  |  | 0.636^2b^ |  |
| Female | 73 (58%) | 32 (60%) | 41 (56%) |  |  |
| Male | 53 (42%) | 21 (40%) | 32 (44%) |  |  |
| **ADHD medication**^1b^ |  |  |  | 0.454^2b^ |  |
| Unmedicated | 50 (40%) | 19 (36%) | 31 (42%) |  |  |
| Medicated | 76 (60%) | 34 (64%) | 42 (58%) |  |  |
| **BMI** (kg/m^2^) ^1c^ | -0.064 (0.011) | -0.063 (0.012) | -0.066(0.010) | 0.145^2c^ |  |
| **Obesity** (BMI ≥ 30 kg/m2)^1b^ | 28 (22%) | 15 (28%) | 13 (18%) | 0.175^2b^ |  |
| **Age**^1a^ | 63 (43, 78) | 65 (44, 78) | 62 (42, 78) | 0.689^2a^ |  |
| **Psychotropic medication**^1b^ | 45 (36%) | 27 (51%) | 18 (25%) | **0.002**^2b^ |  |
| **Tobacco user**^1b^ | 60 (48%) | 27 (51%) | 33 (45%) | 0.524^2b^ |  |
| **Vegan/Vegetarian**^1b^ | 7 (5.6%) | 5 (9.4%) | 2 (2.7%) | 0.130^2c^ |  |
| ^1a^ Median (IQR); ^1b^ n (%); ^1c^ Mean (SD) | | |  |  |  |
| ^2a^ Wilcoxon rank sum test; ^2b^ Pearson's Chi-squared test (% of total column); ^2c^ Welch Two Sample t-test | | | | |  |

| **Supplementary Table 4b. Micro and Macronutrients.** Nutrients were extracted from an onlone protocol (Frankfurt) and two version sof a food frequency questionnaires (Budapest and Barcelona) and were thus not compared statistically for the overall group. | | | | | | |  |
| --- | --- | --- | --- | --- | --- | --- | --- |
|  |  |  |  |  |  |  |  |
| **Nutrition** | **N** | **Overall** | **HIP** | **LIP** | **p-value**^1^ | **recommended vales**^2^ |  |
|  |  | **N = 126** | **N = 53** | **N = 73** |  |  |  |
| **Total energy intake** (kcal/d) | | | | | | 2000 |  |
| Barcelona | 37 | 1,756 (1,440, 2,000) | 1,634 (1,561, 1,818) | 1,787 (1,409, 2,038) | 0.821 |  |  |
| Budapest | 32 | 2,075 (1,797, 2,404) | 1,612 (1,612, 1,612) | 2,114 (1,849, 2,436) | 0.194 |  |  |
| Frankfurt | 33 | 2,338 (1,856, 2,530) | 2,215 (1,814, 2,513) | 2,530 (2,434, 2,605) | 0.288 |  |  |
| **Carbohydrates** (g/d) | | | | | | 130 |  |
| Barcelona | 37 | 180 (163, 197) | 173 (171, 180) | 183 (153, 213) | 0.226 |  |  |
| Budapest | 32 | 247 (201, 266) | 177 (177, 177) | 248 (211, 266) | 0.194 |  |  |
| Frankfurt | 33 | 234 (189, 277) | 235 (186, 275) | 211 (208, 284) | 0.745 |  |  |
| **Proteins** (g/d) | | | | | | 50 |  |
| Barcelona | 37 | 85 (72, 97) | 88 (82, 92) | 85 (66, 101) | 0.566 |  |  |
| Budapest | 32 | 80 (61, 100) | 57 (57, 57) | 82 (65, 101) | 0.386 |  |  |
| Frankfurt | 33 | 77 (57, 103) | 75 (53, 88) | 103 (96, 137) | 0.054 |  |  |
| **Fat** (g/d) | | | | | | 78 |  |
| Barcelona | 37 | 69 (57, 85) | 66 (57, 85) | 70 (58, 83) | 0.848 |  |  |
| Budapest | 32 | 85 (73, 115) | 74 (74, 74) | 85 (73, 115) | 0.448 |  |  |
| Frankfurt | 33 | 103 (85, 125) | 104 (80, 126) | 99 (95, 106) | 0.930 |  |  |
| **Monosaturated fatty acids** (g/d) | | | | | | 33-44 |  |
| Barcelona | 37 | 14 (9, 18) | 16 (12, 17) | 13 (7, 19) | 0.412 |  |  |
| Budapest | 32 | 26 (21, 31) | 21 (21, 21) | 26 (22, 32) | 0.330 |  |  |
| Frankfurt | 33 | 35 (28, 45) | 36 (28, 45) | 35 (32, 38) | 0.977 |  |  |
| **Polyunsaturated fatty acids** (g/d) | | | | | | 15 |  |
| Barcelona | 37 | 5.5 (4.2, 10.4) | 7.7 (5.1, 11.6) | 5.0 (3.9, 7.8) | 0.119 |  |  |
| Budapest | 32 | 22 (16, 27) | 21 (21, 21) | 23 (16, 27) | 0.914 |  |  |
| Frankfurt | 33 | 15 (10, 20) | 15 (9, 20) | 21 (17, 22) | 0.288 |  |  |
| **Saturated fatty acids** (g/d) | | | | | | 20 |  |
| Barcelona | 37 | 23 (16, 35) | 22 (21, 26) | 25 (15, 35) | 0.794 |  |  |
| Budapest | 32 | 26 (21, 34) | 23 (23, 23) | 26 (20, 35) | 0.745 |  |  |
| Frankfurt | 33 | 42 (26, 51) | 42 (24, 55) | 42 (37, 43) | 0.883 |  |  |
| **Alpha-linolenic Acid** (g/d) | | | | | | 1.1-1.6 |  |
| Barcelona | 36 | 0.44 (0.25, 0.69) | 0.53 (0.42, 1.30) | 0.40 (0.21, 0.61) | 0.154 |  |  |
| Budapest | 32 | 0.81 (0.56, 1.07) | 0.51 (0.51, 0.51) | 0.82 (0.58, 1.08) | 0.330 |  |  |
| Frankfurt | NA | NA | NA | NA | NA |  |  |
| **Eicosapentaenoic Acid** (g/d) | | | | | | 1 |  |
| Barcelona | 37 | 0.02 (0.00, 0.05) | 0.02 (0.01, 0.04) | 0.01 (0.00, 0.06) | 0.602 |  |  |
| Budapest | 32 | 0.00 (0.00, 0.02) | 0.00 (0.00, 0.00) | 0.00 (0.00, 0.02) | 0.248 |  |  |
| Frankfurt | 33 | 0.02 (0.01, 0.12) | 0.02 (0.01, 0.13) | 0.04 (0.03, 0.08) | 0.569 |  |  |
| **Docosahexaenoic Acid** (g/d) | | | | | | 1 |  |
| Barcelona | 37 | 0.04 (0.01, 0.11) | 0.11 (0.03, 0.12) | 0.04 (0.01, 0.09) | 0.318 |  |  |
| Budapest | 32 | 0.00(0.00, 0.00) | 0.00(0.00, 0.00) | 0.00(0.00, 0.00) | >0.999 |  |  |
| Frankfurt | 33 | 0.07 (0.02, 0.24) | 0.06 (0.02, 0.22) | 0.24 (0.19, 0.30) | 0.176 |  |  |
| **Omega 3** (g/d) | | | | | | 1.1-1.6 |  |
| Barcelona | 37 | 0.60 (0.40, 1.04) | 0.69 (0.57, 1.42) | 0.54 (0.38, 0.92) | 0.209 |  |  |
| Budapest | 32 | 0.83 (0.57, 1.34) | 0.51 (0.51, 0.51) | 0.83 (0.58, 1.35) | 0.330 |  |  |
| Frankfurt | 31 | 1.50 (0.90, 2.34) | 1.41 (0.86, 2.31) | 1.73 (1.59, 2.07) | 0.593 |  |  |
| **Omega 6** (g/d) | | | | | | 15 |  |
| Barcelona | 37 | 4.4 (3.4, 5.2) | 4.8 (4.4, 10.1) | 3.9 (2.8, 5.0) | **0.038 *** |  |  |
| Budapest | 32 | 20.2 (15.5, 24.3) | 20.6 (20.6, 20.6) | 20.0 (15.2, 24.3) | 0.952 |  |  |
| Frankfurt | 29 | 9.4 (6.3, 13.0) | 9.2 (6.3, 11.8) | 17.2 (11.8, 17.8) | 0.317 |  |  |
| **Omega3/Omega 6 Ratio** | | | | | | 2 |  |
| Barcelona | 37 | 6.78 (5.60, 8.22) | 7.02 (6.77, 8.14) | 6.78 (5.48, 8.28) | 0.614 |  |  |
| Budapest | 32 | 23 (18, 30) | 40 (40, 40) | 23 (17, 29) | 0.256 |  |  |
| Frankfurt | 31 | 6.76 (4.62, 8.49) | 6.43 (4.66, 8.48) | 7.59 (5.96, 8.77) | 0.826 |  |  |
| **Fiber** (g/d) | | | | | | 25-38 |  |
| Barcelona | 37 | 13 (8, 18) | 13 (11, 15) | 14 (8, 18) | 0.931 |  |  |
| Budapest | 32 | 21 (16, 24) | 17 (17, 17) | 21 (16, 24) | 0.745 |  |  |
| Frankfurt | 32 | 21 (14, 32) | 22 (14, 31) | 21 (17, 36) | 0.952 |  |  |
| **Cholesterol** (mg/d) | | | | | | 300 |  |
| Barcelona | 37 | 216 (146, 311) | 264 (190, 372) | 197 (134, 309) | 0.154 |  |  |
| Budapest | 32 | 306 (207, 385) | 174 (174, 174) | 310 (231, 386) | 0.330 |  |  |
| Frankfurt | 33 | 330 (176, 401) | 323 (118, 400) | 362 (338, 480) | 0.317 |  |  |
| **Magnesium** (mg/d) | | | | | | 310-420 |  |
| Barcelona | 36 | 135 (94, 172) | 135 (110, 172) | 134 (89, 171) | 0.494 |  |  |
| Budapest | 32 | 377 (334, 485) | 413 (413, 413) | 373 (332, 492) | 0.828 |  |  |
| Frankfurt | 27 | 347 (246, 430) | 346 (243, 443) | 371 (361, 380) | 0.689 |  |  |
| **Zinc** (mg/d) | | | | | | 10 |  |
| Barcelona | 37 | 4.71 (3.04, 7.71) | 5.61 (4.58, 7.27) | 4.10 (2.82, 7.86) | 0.367 |  |  |
| Budapest | 32 | 7.73 (6.58, 10.10) | 7.55 (7.55, 7.55) | 7.79 (6.48, 10.12) | 0.914 |  |  |
| Frankfurt | 33 | 10.6 (8.5, 15.0) | 10.2 (8.4, 14.3) | 19.2 (14.9, 19.5) | 0.087 |  |  |
| **Iron** (mg/d) | | | | | | 12 |  |
| Barcelona | 37 | 9.8 (6.5, 11.4) | 10.4 (10.3, 10.6) | 8.9 (6.2, 11.4) | 0.188 |  |  |
| Budapest | 32 | 10.3 (8.3, 12.1) | 6.1 (6.1, 6.1) | 10.7 (8.4, 12.2) | 0.159 |  |  |
| Frankfurt | 33 | 13 (10, 18) | 13 (9, 17) | 17 (14, 21) | 0.382 |  |  |
| **Copper** (μg/d) | | | | | | 900 |  |
| Barcelona | 37 | 0.58 (0.33, 0.73) | 0.62 (0.47, 0.73) | 0.57 (0.32, 0.74) | 0.348 |  |  |
| Budapest | 32 | 1,008 (715, 1,275) | 658 (658, 658) | 1,028 (734, 1,295) | 0.386 |  |  |
| Frankfurt | 33 | 1,811 (1,177, 2,347) | 1,592 (1,158, 2,367) | 2,200 (2,018, 2,223) | 0.453 |  |  |
| **Sodium** (mg/d) | | | | | | 2300 |  |
| Barcelona | 36 | 2,933 (2,535, 4,197) | 2,973 (2,639, 4,116) | 2,894 (2,512, 4,212) | 0.914 |  |  |
| Budapest | 32 | 3,823 (2,910, 5,051) | 3,280 (3,280, 3,280) | 4,016 (2,845, 5,061) | 0.745 |  |  |
| Frankfurt | 33 | 2,460 (1,876, 3,711) | 2,437 (1,889, 3,687) | 2,736 (2,226, 4,370) | 0.745 |  |  |
| **Potassium** (mg/d) | | | | | | 2600-3400 |  |
| Barcelona | 37 | 1,376 (1,070, 1,658) | 1,533 (1,213, 1,649) | 1,279 (1,004, 1,664) | 0.373 |  |  |
| Budapest | 32 | 2,864 (2,346, 3,329) | 2,427 (2,427, 2,427) | 2,867 (2,274, 3,342) | 0.448 |  |  |
| Frankfurt | 33 | 3,087 (2,423, 3,697) | 3,076 (2,372, 3,746) | 3,119 (2,797, 3,251) | 0.930 |  |  |
| **Vitamin D** (μg/d) | | | | | | 15 |  |
| Barcelona | 37 | 1.98 (0.84, 3.80) | 1.98 (0.75, 2.34) | 2.54 (0.90, 4.06) | 0.436 |  |  |
| Budapest | 32 | 2.07 (1.57, 3.50) | 0.93 (0.93, 0.93) | 2.11 (1.63, 3.55) | 0.159 |  |  |
| Frankfurt | 33 | 1.94 (0.86, 3.41) | 2.00 (0.82, 3.48) | 1.94 (1.92, 2.32) | 0.930 |  |  |
| **Vitamin E** (mg/d) | | | | | | 12 |  |
| Barcelona | 37 | 3.5 (2.4, 4.7) | 3.3 (3.1, 3.6) | 3.8 (2.0, 5.2) | 0.638 |  |  |
| Budapest | 32 | 15.0 (11.6, 17.7) | 13.6 (13.6, 13.6) | 15.2 (11.4, 17.8) | 0.665 |  |  |
| Frankfurt | 33 | 12 (9, 17) | 12 (9, 17) | 12 (12, 15) | 0.701 |  |  |
| **Vitamin K** (mg/d) | | | | | | 75-120 |  |
| Barcelona | 37 | 29 (20, 52) | 34 (20, 41) | 28 (19, 53) | 0.741 |  |  |
| Budapest | 32 | 69 (57, 95) | 18 (18, 18) | 70 (60, 98) | 0.104 |  |  |
| Frankfurt | 29 | 75 (47, 118) | 74 (45, 113) | 116 (96, 190) | 0.196 |  |  |
| **Vitamin B6** (mg/d) | | | | | | 1.3-1.7 |  |
| Barcelona | 37 | 0.93 (0.64, 1.15) | 1.03 (0.96, 1.18) | 0.89 (0.62, 1.12) | 0.096 |  |  |
| Budapest | 32 | 1.70 (1.35, 2.02) | 1.70 (1.70, 1.70) | 1.70 (1.34, 2.03) | >0.999 |  |  |
| Frankfurt | 33 | 1.64 (1.27, 2.23) | 1.69 (1.22, 2.20) | 1.41 (1.36, 2.02) | 0.876 |  |  |
| **Vitamin B12** (μg/d) | | | | | | 2 |  |
| Barcelona | 36 | 2.67 (1.54, 3.99) | 3.20 (2.23, 3.74) | 2.59 (1.34, 4.00) | 0.391 |  |  |
| Budapest | 32 | 2.70 (1.91, 3.52) | 2.03 (2.03, 2.03) | 2.70 (1.90, 3.68) | 0.516 |  |  |
| Frankfurt | 27 | 2.81 (1.32, 4.38) | 2.45 (1.15, 4.39) | 3.54 (3.04, 3.98) | 0.511 |  |  |
| **Vitamin A** (μg/d) | | | | | | 700-900 |  |
| Barcelona | 37 | 174 (87, 260) | 127 (75, 174) | 185 (95, 306) | 0.188 |  |  |
| Budapest | 32 | 333 (221, 546) | 159 (159, 159) | 335 (227, 547) | 0.159 |  |  |
| Frankfurt | 33 | 956 (600, 1,288) | 989 (540, 1,303) | 906 (803, 1,056) | 0.883 |  |  |
| **Folate** (μg/d) | | | | | | 400 |  |
| Barcelona | 37 | 48 (22, 65) | 39 (6, 54) | 51 (23, 70) | 0.296 |  |  |
| Budapest | 32 | 146 (108, 188) | 58 (58, 58) | 50 (112, 190) | 0.104 |  |  |
| Frankfurt | 33 | 246 (202, 343) | 244 (204, 343) | 260 (211, 399) | 0.883 |  |  |
| **Tryptophan** (mg/d) | | | | | | 250-425 |  |
| Barcelona | 37 | 0.14 (0.06, 0.24) | 0.19 (0.12, 0.28) | 0.12 (0.05, 0.23) | 0.280 |  |  |
| Budapest | 32 | 877 (688, 1,203) | 829 (829, 829) | 901 (671, 1,207) | 0.938 |  |  |
| Frankfurt | 33 | 897 (660, 1,332) | 866 (626, 1,032) | 1,332 (1,203, 1,560) | 0.054 |  |  |
| **Alcohol (g/d)** | | | | | | 0 |  |
| Barcelona | 37 | 0 (0, 6) | 0 (0, 4) | 0 (0, 7) | 0.852 |  |  |
| Budapest | 32 | 0.00 (0.00, 2.79) | 0.00 (0.00, 0.00) | 0.00 (0.00, 2.92) | 0.553 |  |  |
| Frankfurt | 33 | 0.4 (0.0, 4.1) | 0.5 (0.0, 3.9) | 0.0 (0.0, 17.1) | 0.635 |  |  |
| All values displayed as Median (IQR). Recommended values are based on National Institutes of Health Office of Dietary Supplements. Nutrient Recommendations. https://ods.od.nih.gov/HealthInformation/nutrientrecommendations.aspx#dri | | | | | | |  |
| ^1^ Wilcoxon rank sum test | |  |  |  |  |  |  |
| ^2^ ranges display values varying between females and males | | | |  |  |  |  |

| **Supplementary Table 5.** Post-hoc ANOVA results for comparison of unmedicated vs. MPH, LDX medicated ADHD participants. | | | | |  |
| --- | --- | --- | --- | --- | --- |
| **Cluster** | **Protein** | **post-hoc ANOVA** | | |  |
|  |  |  |  |  |  |
|  |  | **F** | **p-value** | **p.adj^1^** |  |
| **Cluster 6** | **ADA** | 0.379 | 0.685 | 0.723 |  |
|  | **CD40** | 0.441 | 0.644 | 0.723 |  |
|  | **SIRT2** | 1.901 | 0.154 | 0.723 |  |
|  | **AXIN1** | 2.313 | 0.104 | 0.723 |  |
|  | **STAMBP** | 1.941 | 0.148 | 0.723 |  |
|  | **CD244** | 0.457 | 0.635 | 0.723 |  |
|  | **PD-L1** | 0.577 | 0.563 | 0.723 |  |
|  | **EIF4EBP1** | 0.74 | 0.479 | 0.723 |  |
|  | **CASP8** | 0.562 | 0.571 | 0.723 |  |
|  | **TNFSF14** | 1.7 | 0.187 | 0.723 |  |
|  | **ST1A1** | 0.277 | 0.758 | 0.758 |  |
|  | **CCL3** | 1.554 | 0.216 | 0.723 |  |
|  | **CCL4** | 0.687 | 0.505 | 0.723 |  |
|  | **MCP-2** | 0.542 | 0.583 | 0.723 |  |
|  | **LAPTGF β-1** | 2.003 | 0.14 | 0.723 |  |
|  | **CXCL6** | 0.754 | 0.473 | 0.723 |  |
|  | **MCP-4** | 0.86 | 0.426 | 0.723 |  |
|  | **CXCL11** | 0.716 | 0.491 | 0.723 |  |
|  | **CXCL5** | 0.461 | 0.632 | 0.723 |  |
|  | **CXCL1** | 0.371 | 0.691 | 0.723 |  |
|  | **IL-8** | 1.157 | 0.318 | 0.723 |  |
|  | **IL-7** | 0.526 | 0.592 | 0.723 |  |
|  | **MMP1** | 1.025 | **0.362** | 0.723 |  |
| ^1^ Benjamini & Hochberg correction for multiple testing | | | | |  |

| **Supplementary Table 6.** MANOVA and post-hoc ANOVA results for the effects of psycho-tropic medication in combination with ADHD medication. | | | | | | | | |  |
| --- | --- | --- | --- | --- | --- | --- | --- | --- | --- |
| **Cluster** | **Protein** | **Psychotrophic medicated** | | **Psychotrophic unmedicated** | | **Psychotrophic medicated vs unmedicated** | | |  |
|  |  | **N = 45** | | **N = 81** | |  |  |  |  |
|  |  | **mean** | **sd** | **mean** | **sd** | **F^1^** | **p-value^1^** | **p.adj^2^** |  |
| **Cluster 2** | **DNER** | 8.4 | 0.26 | 8.42 | 0.24 | 0.306 | 0.581 | 0.756 |  |
|  | **uPA** | 9.51 | 0.23 | 9.45 | 0.27 | 1.643 | 0.202 | 0.376 |  |
|  | **TWEAK** | 8.56 | 0.27 | 8.56 | 0.27 | 0.007 | 0.936 | 0.936 |  |
|  | **CST5** | 6.15 | 0.49 | 6 | 0.42 | 3.259 | 0.073 | 0.239 |  |
|  | **OPG** | 9.82 | 0.28 | 9.68 | 0.31 | 6.517 | **0.012** | 0.077 |  |
|  | **CCL28** | 2.35 | 0.39 | 2.22 | 0.34 | 4.11 | **0.045** | 0.194 |  |
|  | **MMP-10** | 9.08 | 0.84 | 8.89 | 0.65 | 1.962 | 0.164 | 0.355 |  |
|  | **TNFB** | 4.59 | 0.36 | 4.58 | 0.39 | 0.021 | 0.884 | 0.936 |  |
|  | **LIF-R** | 3.71 | 0.23 | 3.75 | 0.2 | 0.7 | 0.404 | 0.584 |  |
|  | **Flt3L** | 8.8 | 0.36 | 8.56 | 0.4 | 12.182 | **0.001** | **0.009** |  |
|  | **CX3CL1** | 3.83 | 0.41 | 3.86 | 0.33 | 0.217 | 0.642 | 0.759 |  |
|  | **FGF-19** | 7.9 | 0.76 | 7.65 | 0.94 | 2.21 | 0.14 | 0.355 |  |
| **Cluster 4** | **CCL25** | 5.9 | 0.52 | 5.66 | 0.46 | 7.119 | **0.009** | **0.028** |  |
|  | **CDCP1** | 2.5 | 0.68 | 1.98 | 0.45 | 27.508 | **< 0.001** | **< 0.001** |  |
|  | **IL18** | 8.65 | 0.59 | 8.41 | 0.54 | 5.217 | **0.024** | **0.045** |  |
|  | **MCP-1** | 10.64 | 0.37 | 10.52 | 0.37 | 3.246 | 0.074 | 0.12 |  |
|  | **CCL11** | 7.4 | 0.56 | 7.21 | 0.37 | 5.669 | **0.019** | **0.041** |  |
|  | **TRAIL** | 7.63 | 0.33 | 7.55 | 0.28 | 1.806 | 0.181 | 0.236 |  |
|  | **IL-10RB** | 5.91 | 0.3 | 5.78 | 0.25 | 6.567 | **0.012** | **0.03** |  |
|  | **CD5** | 5.02 | 0.28 | 4.99 | 0.29 | 0.276 | 0.6 | 0.6 |  |
|  | **VEGFA** | 10.69 | 0.41 | 10.48 | 0.32 | 10.298 | **0.002** | **0.011** |  |
|  | **CSF-1** | 9.75 | 0.21 | 9.72 | 0.19 | 0.73 | 0.394 | 0.466 |  |
|  | **TNF** | 2.21 | 0.32 | 2.12 | 0.34 | 2.068 | 0.153 | 0.221 |  |
|  | **IL-15RA** | 0.99 | 0.17 | 0.93 | 0.08 | 7.421 | **0.007** | **0.028** |  |
|  | **SLAMF1** | 2.18 | 0.44 | 2.14 | 0.28 | 0.355 | 0.552 | 0.598 |  |
| ^1^ post-hoc ANOVA ^2^ Benjamini & Hochberg correction for multiple testing | | | | | | | | |  |
|  |  |  |  |  |  |  |  |  |  |

**Sample Size calculation**

A priori analysis

**F tests -** ANOVA: Fixed effects, omnibus, one-way

**Analysis:** A priori: Compute required sample size

**Input:** Effect size f = 0.25

α err prob = 0.05

Power (1-β err prob) = 0.80

Number of groups = 2

**Output:** Noncentrality parameter λ = 8.0000000

Critical F = 3.9163246

Numerator df = 1

Denominator df = 126

Total sample size = 128

Actual power = 0.8014596

With achieved sample size- 126

[3] *-- Tuesday, November 07, 2023 -- 12:06:30*

**F tests -** ANOVA: Fixed effects, omnibus, one-way

**Analysis:** Post hoc: Compute achieved power

**Input:** Effect size f = 0.25

α err prob = 0.05

Total sample size = 126

Number of groups = 2

**Output:** Noncentrality parameter λ = 7.8750000

Critical F = 3.9175498

Numerator df = 1

Denominator df = 124

Power (1-β err prob) = 0.7951683
